# In-Depth Theoretical Investigations of Borazine’s Aromaticity: Tailoring Electron Delocalization through Substituent Effects

**DOI:** 10.3390/molecules29204902

**Published:** 2024-10-16

**Authors:** Alex-Cristian Tomut, Ionut-Tudor Moraru, Gabriela Nemes

**Affiliations:** Faculty of Chemistry and Chemical Engineering, Department of Chemistry, Babeș-Bolyai University, 1 M. Kogalniceanu Street, 400084 Cluj-Napoca, Romania; alex.tomut@stud.ubbcluj.ro

**Keywords:** “inorganic” benzene, aromaticity, DFT methods, aromaticity indices, NBO analyses, hyperconjugations, Pauli repulsions

## Abstract

The current study investigates the influence of several R substituents (e.g., Me, SiH_3_, F, Cl, Br, OH, NH_2_, etc.) on the aromaticity of borazine, also known as the “inorganic benzene”. By performing hybrid DFT methods, blended with several computational techniques, e.g., Natural Bond Orbital (NBO), Quantum Theory of Atoms in Molecules (QTAIM), Gauge-Including Magnetically Induced Current (GIMIC), Nucleus-Independent Chemical Shift (NICS), and following a simultaneous evaluation of four different aromaticity indices (para-delocalization index (PDI), multi-centre bond order (MCBO), ring current strength (RCS), and NICS parameters), it is emphasized that the aromatic character of B-substituted (B_3_R_3_N_3_H_3_) and N-substituted (B_3_H_3_N_3_R_3_) borazine derivatives can be tailored by modulating the electronic effects of R groups. It is also highlighted that the position of R substituents on the ring structure is crucial in tuning the aromaticity. Systematic comparisons of calculated aromaticity index values (i.e., via regression analyses and correlation matrices) ensure that the reported trends in aromaticity variation are accurately described, while the influence of different R groups on electron delocalization and related aromaticity phenomena is quantitatively assessed based on NBO analyses. The most relevant interactions impacting the aromatic character of investigated systems are (i) the electron conjugations occurring between the p lone pair electrons (LP) on the F, Cl, Br, O or N atoms, of R groups, and the π*(B=N) orbitals on the borazine ring (i.e., LP(R)→π*(B=N) donations), and (ii) the steric-exchange (Pauli) interactions between the same LP and the π(B=N) bonds (i.e., LP(R)↔π(B=N) repulsions), while inductive/field effects influence the aromaticity of the investigated trisubstituted borazine systems to a much lesser extent. This work highlights that although the aromatic character of borazine can be enhanced by grafting electron-donor substituents (F, OH, NH_2_, O^−^, NH^−^) on the N atoms, the stabilization due to aromaticity has only a moderate impact on these systems. By replacing the H substituents on the B atoms with similar R groups, the aromatic character of borazine is decreased due to strong exocyclic LP(R)→π*(B=N) donations affecting the delocalization of π-electrons on the borazine ring.

## 1. Introduction

Since the discovery of benzene’s structure, i.e., a planar hexagonal ring with equal bond lengths between carbon atoms, or its special physical/chemical properties, i.e., predilection towards substitution reactions, unexpected chemical inertness, distinctive ^1^H-NMR chemical shifts of the H atoms attached to the ring, and so forth, chemists around the world have been eagerly trying to develop models that accurately account for these peculiar features. Therefore, aromaticity has become a core concept in modern organic chemistry [1,2,3]. The first chemical formulas of benzene were proposed by Kekulé more than 150 years ago [4]. Following the development of quantum theory, further clarifications were brought into its electronic structure, with Hückel’s model [5,6] or Robinson’s sextet rule [7] becoming key concepts that are currently used in assessing and explaining the aromaticity of organic compounds.

Even though aromatic motifs are found in copious amounts within organic compounds, this is not the case for inorganic analogues. The synthesis and isolation of inorganic aromatic rings has always been a great challenge for chemists around the world. Stock and Pohland were the first to obtain a stable benzene-like compound which does not contain carbon atoms. This molecule, known in the literature as borazine, has the B_3_H_6_N_3_ formula and is frequently regarded as the “inorganic benzene” [8]. Other heavier counterparts of borazine were synthesized over the years, with Power’s group probably being the most active in this field. In their synthetic approaches, they commonly employ bulky substituents to kinetically stabilize ring structures with the B_3_P_3_, Ge_3_N_3_, etc., skeleton [9,10,11,12,13]. During the past decade, the synthesis of a five-membered [P_2_N_3_]^−^ aromatic ring was reported by Velian and Cummins [14]. The solid-state structure of this anion has been characterized, while complementary computational studies confirmed its aromatic character [14]. The most recent work reporting novel benzene-like inorganic rings was published by Seitz et al. in 2016 [15], in which they successfully isolated and fully characterized several six- and four-membered silicon–phosphorus and silicon–arsenic ring structures.

Quantum chemical calculations are currently employed on inorganic benzenes, either in purely theoretical studies [16,17,18] or as complementary tools, in order to allow for a better understanding of experimental findings [14,15]. In both cases, the main goal is to understand the electron delocalization phenomena and to evaluate the stability of these compounds. However, as already pointed out, the synthesis of such heavier analogues of benzene is inherently hard, due to their lack of stability. This suggests that the aromatic character of these compounds is either very low or non-existent, or it does not stabilize the inorganic ring system enough. Nyulászi et al. reported that silicon–nitrogen benzene-like structures are more stable in the silylenic form (i.e., as (HNSi)_3_ species) than as (HSiN)_3_ rings [16]. Yet, according to their calculations, the increased thermal stabilization of the former is not necessarily related to aromaticity, but rather to the increased stability of the N-H bonds compared to the Si-H ones. Other computational studies of Wu and coworkers involved QTAIM and electron localization function (ELF) topological analyses and suggested that the π-electron density has only a small influence on the bonding between atoms in the cycle [17]. The most recent theoretical work of Shaik et al. was carried out on several E_3_N_3_H_3_ model systems (E = C, Si, Ge, Sn, Pb) [18]. Based on Valence Bond theory (VBT) calculations, they reported that E-N bonds have a high charge-shift character, such inorganic counterparts not being aromatic like benzene, but rather stabilized by charge-shift resonance energies.

Regarding borazine, to gain insight into its aromaticity, the H atoms were replaced by different types of substituents that induce both electron-donating and -withdrawing effects, with the aim of modulating electron delocalization. According to the literature [19,20,21,22], by replacing the H groups on the B atoms with common substituents, the general tendency is to obtain compounds with a lower aromatic character, except for the substituents that allow for an extended π-conjugation (e.g., NO_2_ and CN). When replacing the H atoms on the N atoms, the opposite trend was suggested, although other theoretical studies conducted on B_3_H_3_N_3_F_3_ systems by Miao et al. led to different conclusions [23,24]. The latter are supported by an older paper published by Parker [25]. However, these discrepancies can be rooted in the different methods (or simply aromaticity indices) employed in these studies. Owing to these inconsistencies, further clarifications regarding the influence of the substituent effect on the aromaticity of borazine are required, thus allowing for a more comprehensive understanding of underlying phenomena. To the best of our knowledge, there is no quantitative (or at least semi-quantitative) theoretical model that accounts for the variation in aromaticity of substituted borazine rings.

The current study represents an extensive computational investigation that attempts to shed more light on the aromatic character of the “inorganic benzene”, borazine, by combining hybrid DFT methods with four different types of aromaticity indices and NBO techniques. The approach based on direct correlations between data derived from two electronic (PDI and MCBO) and two magnetic (RCS and NICS) indices has not been applied to borazine systems so far. The main goals of this research are to gain a detailed picture of the electron delocalization in substituted borazine rings and to accurately account for the potential impact of the substituent effect on their aromaticity. NBO explorations of secondary electronic (conjugative) effects and steric-exchange (Pauli) interactions allow for a quantitative description of these phenomena.

## 2. Results and Discussions

### 2.1. Model Structures—Geometrical and Vibrational Features

**Figure 1:**
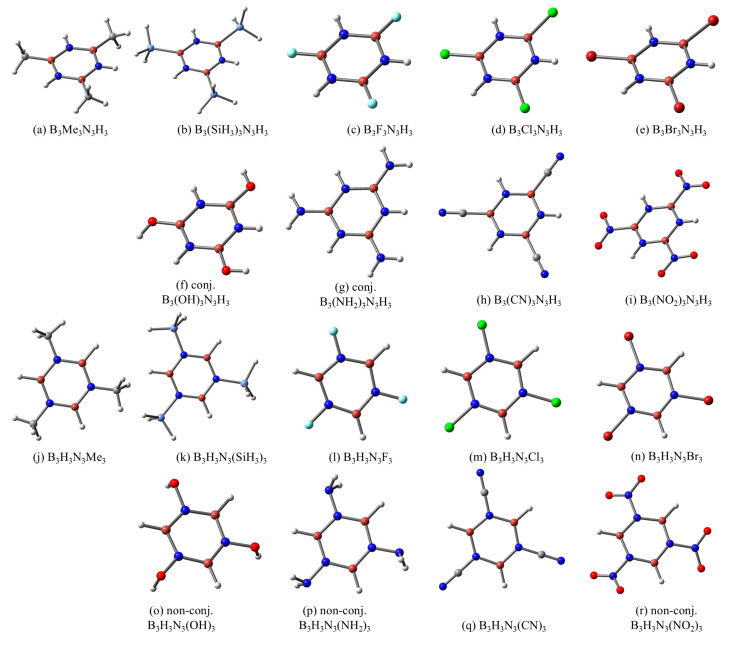
Equilibrium geometries of the most stable conformations for B_3_R_3_N_3_H_3_ and B_3_H_3_N_3_R_3_ model systems, obtained by selectively replacing the H substituents grafted on the B (**a**–**i**) and on the N (**j**–**r**) atoms with several R substituents (R = Me, SiH_3_, F, Cl, Br, OH, NH_2_, CN, and NO_2_).

Starting from the molecular structure of B_3_N_3_H_6_, several model systems were obtained by replacing the H substituents on the B or N atoms with different R groups that are commonly used in organic chemistry (Figure 1) to induce specific electronic effects in aromatic compounds. In fact, the investigated R substituents can be classified based on their predominant electronic effect on benzene, as follows: Me and SiH_3_ (+I, inductive/field donation); F, Cl, and Br (-I, inductive/field attraction); OH and NH_2_ (+E, mesomeric donation); and CN and NO_2_ (-E, mesomeric attraction). Two types of model systems were systematically assessed: (i) one series in which the hydrogen substituents placed on the boron atoms were replaced by R groups (i.e., the B-substituted models—B_3_R_3_N_3_H_3_), and (ii) another series in which the hydrogens on the nitrogen atoms were replaced by the same R substituents (i.e., the N-substituted models—B_3_H_3_N_3_R_3_). The resulting structures were optimized at the DFT-PBE0/Def2-TZVP level of theory. It was ensured that all obtained structures had the highest symmetry point group. The most relevant geometrical features, vibrational modes, and energy differences are given in the Supplementary Materials (Tables S1–S3 and Figures S5–S7).

By substituting the H atoms on the B atoms of borazine, it results in nine minima, with all of them exhibiting planar structures of the B_3_N_3_ ring. In the case of OH, NH_2_, and NO_2_ groups, several geometries are possible, with the main difference between them being the relative orientation of these substituents with respect to the cyclic B_3_N_3_ backbone. Yet, DFT calculations suggest that the most stable structures correspond to the case where the p lone pair electrons (LP) of O or N atoms are fully conjugated with the ring structure, resulting in a coplanar arrangement of the borazine cycle and the atoms contained in the OH, NH_2_, or NO_2_ groups (Figure 1f,g,i). Regarding the B_3_H_3_N_3_R_3_ systems, their equilibrium geometries reveal many similar features as those of B-substituted counterparts, e.g., planar ring structures, similar B-N bond distances, and so forth. However, several differences are noticed between the N-substituted and the B-substituted derivatives, especially when involving OH, NH_2_, and NO_2_ substituents. These are mainly related to the extended electron conjugation between these groups and the borazine ring, which occurs in the latter (as already pointed out), but not in the case of B_3_H_3_N_3_(OH)_3_, B_3_H_3_N_3_(NH_2_)_3_, and B_3_H_3_N_3_(NO_2_)_3_ models. Thus, the increased repulsion between the LP(O), or LP(N), and the π(B=N) orbitals (preponderantly localized on the N atoms) can possibly explain the preference of such N-substituted model derivatives to adopt equilibrium geometries that lack a planar arrangement between the OH, NH_2_, or NO_2_ groups and the B_3_N_3_ moiety (Figure 1o,p,r).

Thus, for several R substituents (OH, NH_2_, or NO_2_), the conjugation of such groups with the borazine ring can occur (for the B-substituted systems) or not (for the N-substituted models) within the equilibrium geometries of the most stable conformations (Figure 1). The optimization of the counterpart structures, in which the extended conjugation for the B-substituted models is precluded while for N-substituted ones is facilitated, was also performed in this work. According to the vibrational analyses, such structures are higher-order saddle points on the potential energy surface (computed geometries are depicted in Figure S5). Hereafter, model structures containing the OH, NH_2_, or NO_2_ groups are regarded as (i) conjugated systems (i.e., abbreviated as conj.) in which the p LPs on the O or N atoms of the R groups are collinear with the π orbitals on the borazine ring and (ii) non-conjugated systems (i.e., abbreviated as non-conj.) in which case the p LPs on the O or N atoms of the R substituents are orthogonal to the π orbitals of borazine. Even though some of these systems are, as stated, higher-order saddle points, they will prove useful later in the text for the proof of concept.

### 2.2. B_3_R_3_N_3_H_3_ Models—Aromaticity and Electronic Effects

**Figure 2:**
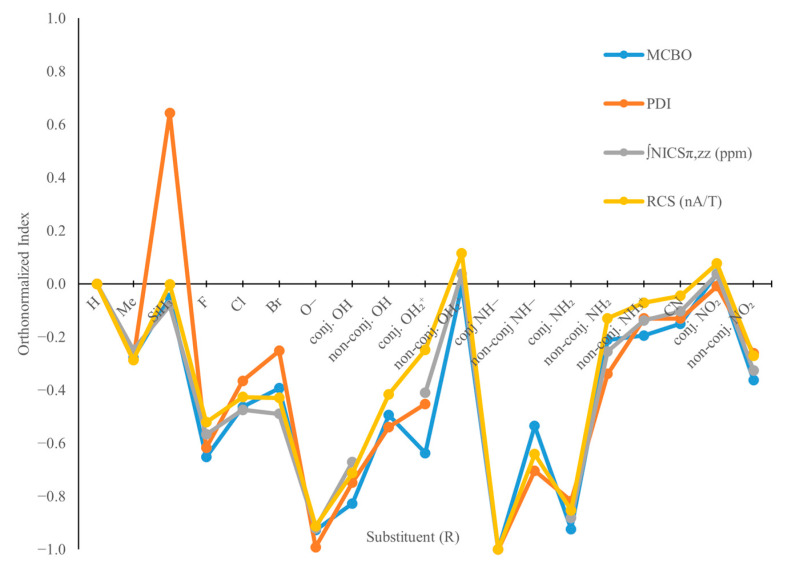
The orthonormalized electronic (MCBO and PDI) and magnetic (∫NICS_π,zz_ and RCS) aromaticity indices computed for the B-substituted borazine model systems, represented with respect to borazine.

The influence of R substituents on borazine’s electron delocalization is assessed by measuring the electronic (MCBO and PDI) and magnetic (NICS and RCS) indices. The computed values of these indices are presented in Table S4, with the most relevant ones being illustrated in Figure 2. Depicted indices for all B_3_R_3_N_3_H_3_ systems were orthonormalized with respect to values computed for B_3_N_3_H_6_, which were set as a reference. The correlation matrix for these indices is given in the Supplementary Materials (Figure S8), showing that no relevant discrepancies are found between them. For most of the B_3_R_3_N_3_H_3_ models, with few exceptions (e.g., R = CN, NO_2_, or SiH_3_), a considerable negative shift of the orthonormalized indices is observed, which indicates a lowering of the aromatic character of these B-substituted borazine. A possible explanation for this behaviour can be expressed in terms of an increased electronegativity of the atoms within R substituents compared to that of B. Therefore, such groups will most probably exhibit inductive and field effects, attracting electrons from their neighbouring B atoms and thus decreasing the electron density on the borazine ring. For the case of the B_3_(SiH_3_)_3_N_3_H_3_ system, where Si has a lower electronegativity than B, the reverse effect is expected—i.e., SiH_3_ groups are likely to act through inductive/field effects that increase the electron density on the B atoms (see Figure S14). 

**Figure 3:**
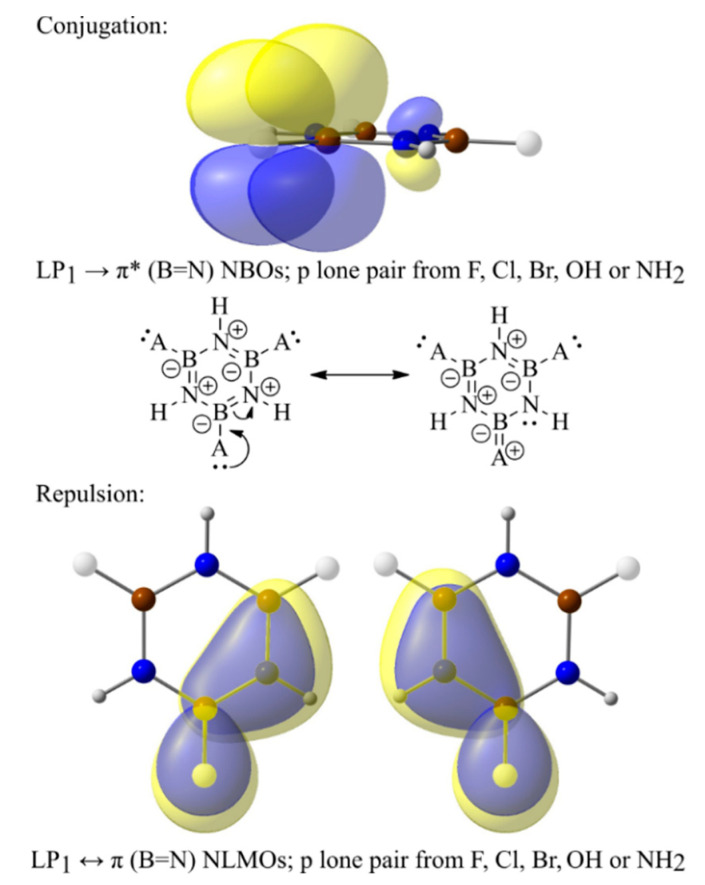
The most relevant secondary electronic effects that impact the aromaticity of B_3_R_3_N_3_H_3_ model systems: conjugation (upper part) and Pauli repulsion (lower part).

Although the electronegativity of R substituents can play an important role in modulating (i.e., basically decreasing) the aromaticity, several other electronic effects should also be taken into account to understand and predict the aromatic character of the borazine-substituted derivatives. An illustrative example in this regard is related to the B_3_R_3_N_3_H_3_ series of -F, -Cl, -Br, -OH, and -NH_2_ substituents. The last two groups have an increased impact on lowering the aromaticity compared to the halogen substituents (Figure 2), albeit their electronegativity is lower (at least compared to that of F). To gain further insights into the electronic structure of B_3_R_3_N_3_H_3_ model derivatives, but also to achieve a better understanding of their aromaticity, NBO calculations were performed on their equilibrium geometries. Based on computed energies, two different types of secondary electronic effects (Figure 3) are relevant: (i) the electron conjugation of a substituent’s p-orbital LPs into π antibonding orbitals on the borazine ring (i.e., LP_1_(R)→π*(B=N), where LP_1_(R) is always a pure p atomic orbital on the F, Cl, Br, O, or N atom of an R substituent), and (ii) Pauli-exchange interactions occurring between the LP_1_(R) and filled π orbitals on the borazine cycle (i.e., LP_1_(R)↔π(B=N) effects).

Both these secondary effects reduce the aromatic character of B_3_R_3_N_3_H_3_ systems. For instance, through LP_1_(R)→π*(B=N) interactions, a significant amount of charge density is donated from the substituents toward the B atoms of borazine. This is because the π*(B=N) orbitals are preponderantly located on boron, which leads to charge localization, resulting in several resonance structures (Figure 3) where aromaticity is not preserved. Particularly for the B_3_(NH_2_)_3_N_3_H_3_ system, LP(N(H_2_))→π*(B=N) interactions are so strong that the B=N conjugated bonds on the ring are significantly weakened, being regarded as merely σ bonds by the NBO analysis, while the exocyclic B-N(H_2_) bonds increase their π character. Therefore, this model structure has not been considered in the following discussions. Besides conjugations, it is shown that LP_1_(R)↔π(B=N) exchange interactions also lead to electron localization, thus decreasing the aromatic character of such borazine derivatives. In fact, the idea that repulsive Pauli interactions could possibly affect the aromaticity of substituted borazine rings was proposed by Baranac-Stojanovic a few years ago [19], but without bringing any quantitative (or at least semi-quantitative) evidence for this claim. Moreover, to the best of our knowledge, there is no other theoretical study addressing this issue on borazine, nor on other aromatic ring structures. Herein, we emphasize for the B_3_R_3_N_3_H_3_ model systems that the LP_1_(R)↔π(B=N) Pauli repulsions impact the electronic distribution on the ring by pushing it further away from the B atoms and leading to an increased density localization on the N atoms. Calculated values of the total conjugation energy (i.e., the sum of all LP_1_(R)→π*(B=N) donations occurring in each system) and total repulsive energy (i.e., the sum of all LP_1_(R)↔π(B=N) exchange interactions occurring in each model structure) are presented in Table 1.

**Figure 4:**
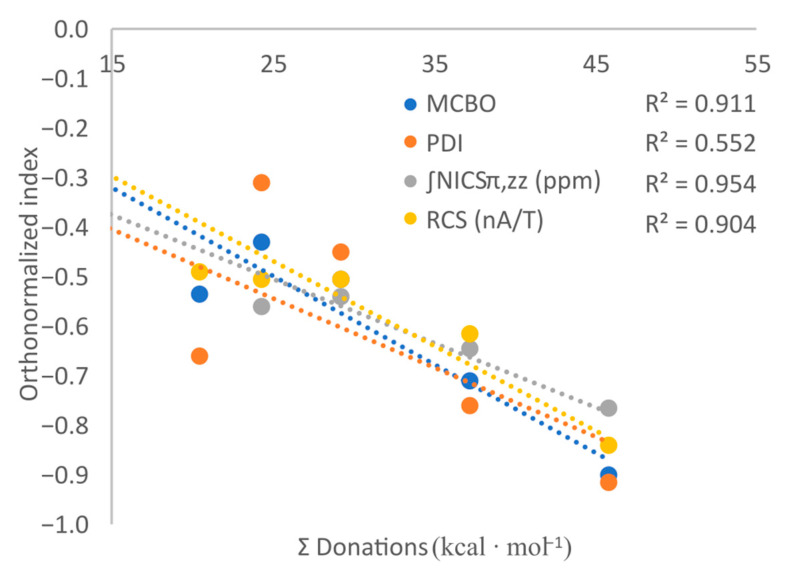
The correlation between the conjugation effects and the aromaticity indices computed for the B_3_R_3_N_3_H_3_ (R = F, Cl, Br, conjugated and non-conjugated OH, and non-conjugated NH_2_) model systems.

By plotting the computed values of investigated indices as a function of the total conjugation (or total repulsion) energy, i.e., (index value)=f(Σ(Donation)) or (index value)=f(Σ(Repulsions)), consistent linear correlations are found in most of the cases. Given that for these systems the conjugations are considerably higher in magnitude than the Pauli repulsions (Table 1), we illustrate in Figure 4 merely the (index value)=f(Σ(Donation)) plot, while correlation matrices between the aromaticity indices and both the total conjugation and repulsion energies are presented in the Supplementary Materials (Figures S10 and S11). It is thus highlighted that increased conjugations or repulsions lower the electronic delocalization, which is yet expected. To gain further insights into the influence of these interactions on the aromaticity of B-substituted borazines, the LP_1_ on the OH or NH_2_ groups of B_3_(OH)_3_N_3_H_3_ and B_3_(NH_2_)_3_N_3_H_3_ models were orthogonalized with respect to the π-system, by rotating the two moieties by 90°. The resulting structures were reoptimized, with their equilibrium geometries being depicted in Figure S5. It is found that the aromatic character of these species is higher compared to that of their conjugated counterparts, but still lower than the one of B_3_N_3_H_6_. This can be explained in terms of donations and repulsions, which, although considerably weaker in the non-conjugated model systems, still have a noticeable contribution, and the inductive/field effects could also contribute to the lowering of the aromatic character (Table 1 and Figure S14).

The aromaticity of B-substituted models containing -E groups can be explained by considering the delocalization of the π electrons on the CN or NO_2_ groups and the inductive/field effects. The computed aromaticity indices for these systems indicate that they are similar in magnitude to those of borazine, meaning that overlaps between the π(B=N) bonds on the ring and the π* orbitals on the CN or NO_2_ substituents are poor. This is confirmed by the low energy values computed for these interactions, e.g., π(B=N)→π*(C≡N) is ca. 2.9 kcal mol^−1^/C≡N group, while π(B=N)→π*(N^+^=O) is <0.1 kcal mol^−1^/NO_2_ group. If the molecular geometry of the B_3_(NO_2_)_3_N_3_H_3_ system is reoptimized so that the NO_2_ moieties are perpendicular to the B_3_N_3_ ring, lower aromaticity indices are computed. This suggests that the inductive/field effects are stronger in this case.

Inductive and field effects also affect the aromatic character of B-substituted borazine systems, but such electronic effects are, in all investigated cases, less impactful than the p→π* conjugations or the p↔π steric-exchange interactions. Hence, their role is not yet fully understood (for further details, see Section 5 in the Supplementary Materials). To shed more light on their influence on aromaticity, we propose an approach that involves the protonation of several R groups (e.g., OH and NH_2_). Thus, a positive charge is induced on these substituents to increase the inductive effect. Another expected outcome of the protonation is to reduce the magnitude of LP_1_(R)→π(B=N) donations, which allows for the isolation of targeted inductive/field effects. The equilibrium structures of [B_3_(OH_2_)_3_N_3_H_3_]^3+^ and [B_3_(NH_3_)_3_N_3_H_3_]^3+^ models are illustrated in the Supplementary Materials, along with selected geometrical data, energetic analyses of the most relevant electronic effects, and the computed aromaticity indices (Tables S2, S3, S4, S6, and S8 and Figures S4, S6, S8, and S13). For the [B_3_(OH_2_)N_3_H_3_]^3+^ model system, the most stable conformation involves the orthogonal displacement between the unprotonated LP at the O atom (i.e., the LP_2_(R), which exhibits a mixed s-/p-orbital character) and the borazine ring. Thus, LP_2_(O)→π*(B=N) conjugations still occur, with the calculated energy being ca. 23.3 kcal mol^−1^/OH_2_^+^ group. The indices computed for this system indicate an increased aromatic character compared to the conjugated B_3_(OH)_3_N_3_H_3_ model. This can be correlated with lower conjugation energies. Still, the aromaticity of [B_3_(OH_2_)N_3_H_3_]^3+^ is lower than that of borazine (Figure 2). By rotating the OH_2_^+^ groups by 90°, followed by reoptimization, LP_2_(O)→π*(B=N) is precluded and the aromaticity of this new system is close to that of borazine. In the case of the [B_3_(NH_3_)_3_N_3_H_3_]^3+^ model, it is found that the aromaticity indices are similar to those computed for the non-conjugated B_3_(NH_2_)_3_N_3_H_3_ system. This suggests that field and inductive effects impact the aromatic character of B-substituted borazines only to a lesser extent. Still, in a comprehensive investigation, it is worth considering such through-space electronic effects.

Similar investigations were also conducted on the [B_3_O_3_N_3_H_3_]^3−^ and [B_3_(NH)_3_N_3_H_3_]^3−^ model derivatives (Figure S7), obtained through the deprotonation of the OH and NH_2_ groups of B_3_OH_3_N_3_H_3_ and B_3_(NH_2_)_3_N_3_H_3_, respectively. According to the calculations, among all investigated B-trisubstituted models, the lowest aromatic character is obtained precisely for these anionic [B_3_O_3_N_3_H_3_]^3−^ and [B_3_(NH)_3_N_3_H_3_]^3−^ systems (see Figure 2, Figures S2, and S4 and Table S4). According to the NBO analyses, these model compounds reveal exocyclic π(B=O) and π(B=N) bonds, which result from very strong LP(R)→π*(B=N) (R = O or N) electron donations, donations that are considerably higher in magnitude than in the neutral counterparts. Thus, evaluation of the conjugation energy was not accessible in the case of the anionic systems, due to technical limitations. The reduced aromatic character of the [B_3_O_3_N_3_H_3_]^3−^ and [B_3_(NH)_3_N_3_H_3_]^3−^ models is thus explained by the localization of electrons at the periphery of the borazine ring.

### 2.3. B_3_H_3_N_3_R_3_ Models—Aromaticity and Electronic Effects

**Figure 5:**
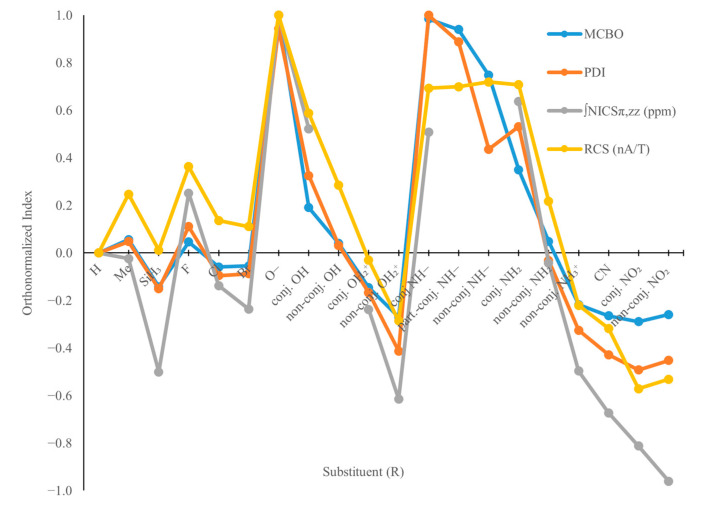
The orthonormalized electronic (MCBO and PDI) and magnetic (∫NICS_π,zz_ and RCS) aromaticity indices of N-substituted borazine models are represented with respect to borazine.

For the N-substituted borazines, i.e., B_3_H_3_N_3_R_3_ systems, the H substituents on the N atoms of borazine were systematically replaced with the same set of R substituents mentioned above (Figure 1j–r). The MCBO, PDI, ∫NICS_π,zz_, and RCS indices were computed for each structure, being presented in the Supplementary Materials along with their correlation matrix (see Table S5 and Figure S9). As in the case of B-substituted borazines, the aromaticity indices computed for the N-substituted models were orthonormalized with respect to values obtained for borazine (Figure 5). Nevertheless, in the case of the B_3_H_3_N_3_R_3_ systems, the R substituents are bound to considerably more electronegative atoms (i.e., according to Pauling’s scale, the electronegativity value for N is 3.04, but only 2.04 for B). Thus, several R groups of B_3_H_3_N_3_R models are expected in several cases to play the role of electron donors, increasing the electronic density on the borazine cycle via inductive and field effects. This could favour the electron delocalization on the ring and consequently modulate the aromatic character of N-substituted borazines.

For the borazine systems substituted with +I groups, calculated indices reveal different trends for the B_3_H_3_N_3_Me_3_ and B_3_H_3_N_3_(SiH_3_)_3_ models (Figure 5). For the former, except for NICS, all indices indicate a slight increase in aromaticity with respect to borazine, while for the latter, most indices suggest the opposite. Although the silicon atom of the SiH_3_ group has a noticeably lower electronegativity than any other investigated substituent, the lower aromaticity of B_3_H_3_N_3_(SiH_3_)_3_ compared to borazine indicates that electron-donating inductive/field effects do not prevail. This can be better understood by comparing the ESP map of borazine with the additive ESP one of the B_3_H_3_N_3_(SiH_3_)_3_ compound (see Figure S15). The differences in aromaticity between B_3_H_3_N_3_Me_3_ and B_3_H_3_N_3_(SiH_3_)_3_ systems can be explained in terms of secondary electronic effects. Even though the π(B=N)→σ*(C-H) donations are stronger than the π(B=N)→σ*(Si-H) ones (i.e., two interactions of ca. 5.8 kcal mol^−1^/CH_3_ substituent compared to two interactions of ca. 4.6 kcal mol^−1^/SiH_3_ group), the additional occurrence of π(B=N)→d(Si) donations (3.1 kcal mol^−1^/SiH_3_) in the latter can further motivate the reduced electron delocalization on the ring. Although the d-orbital contribution to the bond formation of molecules containing heavy p-block elements is often viewed as a computational artifact resulting from the contamination of bonding orbitals with basis set polarization functions [26,27,28,29], this hypothesis has been recently questioned by several theoretical studies [30,31,32]. Thus, it was highlighted that silicon d-orbitals play significant roles in determining the electronic and structural features of siloxanes and silylamines, in line with the current observations.

Concerning the systems involving -I substituents, calculations suggest slightly different trends in the variation of their aromaticity. Computed indices for the B_3_H_3_N_3_F_3_ compound reveal a slight increase in the aromatic character compared to borazine, while for the B_3_H_3_N_3_Cl_3_ and B_3_H_3_N_3_Br_3_ models, only the RCS index suggests a similar tendency. The other indices predict a small decrease in the aromatic character of the last two compounds, which can be motivated in terms of π(B=N)→d(X) (X = Cl or Br) back-donations. Such overlaps are expected to decrease the electron density on the B_3_N_3_ ring, thus impacting electron delocalization. The energy of π(B=N)→d(X) interactions is ca. 5.8 kcal mol^−1^/Cl group for the B_3_H_3_N_3_Cl_3_ system and 4.0 kcal mol^−1^/Br group in the case of the B_3_H_3_N_3_Br_3_ model.

**Figure 6:**
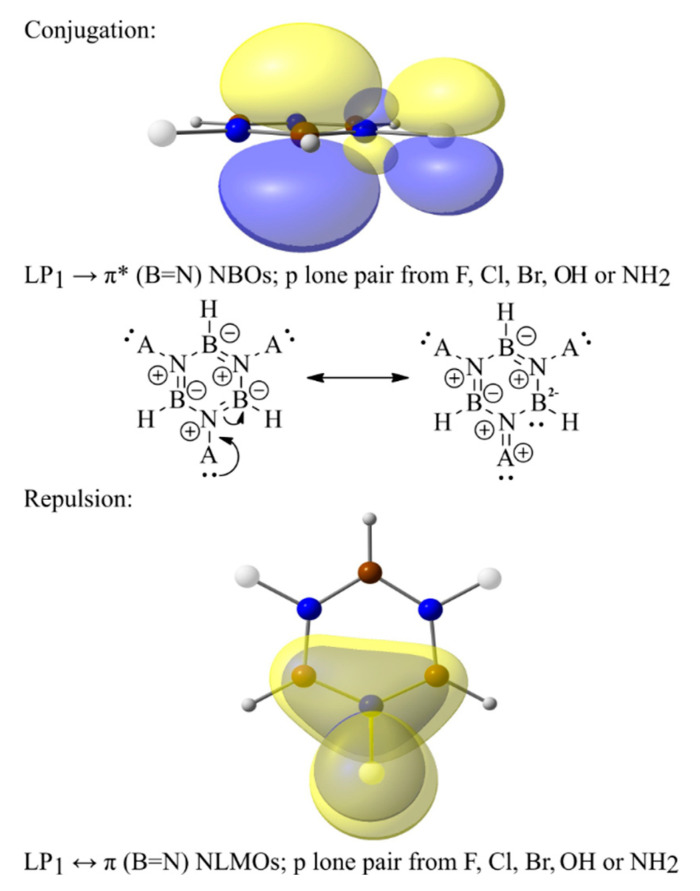
The orthonormalized electronic (MCBO and PDI) and magnetic (∫NICS_π,zz_ and RCS) aromaticity indices of N-substituted borazine models are represented with respect to borazine.

Although substituents that are more electronegative than the N atom are expected to deplete the electron density on the ring, and thus decrease the aromatic character of B_3_H_3_N_3_R_3_ models, calculations reveal the opposite for most of the studied systems. This is also the case for the models substituted with +E groups, for which calculated indices suggest an increase in aromaticity (R = F) or only a slight change (R = OH, NH_2_) (Figure 5). Such behaviour can be explained based on conjugative effects (i.e., LP_1_(R)→π*(B=N) interactions, R = F, Cl, Br, OH, NH_2_) and exchange interactions (i.e., LP_1_(R)↔π(B=N) repulsions), which affect the electron delocalization on the ring (Figure 6). However, the latter is considerably higher in magnitude (Table 2), being, for instance, responsible for the equilibrium geometries of B_3_H_3_N_3_(OH)_3_ and B_3_H_3_N_3_(NH_2_)_3_ systems (i.e., non-conjugated structures; see Figure 1o,p). The OH and NH_2_ groups are oriented in a manner that reduces overlaps between the p-orbital LP_1_ at the O or N atoms and the π(B=N) bonds on the ring, with the two orbitals thus being orthogonal. In this manner, the LP_1_(R)↔π(B=N) repulsions are minimized.

Another consequence of this structural arrangement is that the LP_1_(R)→π*(B=N) conjugations are negligible, especially within B_3_H_3_N_3_(NH_2_)_3_, while for the B_3_H_3_N_3_(OH)_3_ system, the conjugation energy originates mainly from the interactions generated by the other LP (i.e., LP_2_(R) orbital) which exhibits a mixed s/p character. To gain insights into the influence of these effects on aromaticity, the B_3_H_3_N_3_(OH)_3_ and B_3_H_3_N_3_(NH_2_)_3_ structures were reoptimized with several geometrical constraints, so that the Pauli interaction between the LP_1_(R) and the π(B=N) bond would be maximum, resulting in a collinear arrangement of the two orbitals. The equilibrium geometries are illustrated in Figure S5.

Both conjugations and Pauli repulsive electronic effects are present in these systems, yet this time, the steric-exchange interactions are stronger than the LP_1_(R)→π*(B=N) conjugations. This is due to a considerably increased overlap between the LP_1_(R) on the substituents and the π(B=N) bonds, compared to the overlap occurring between the same p LP and π*(B=N) orbitals.

**Figure 7:**
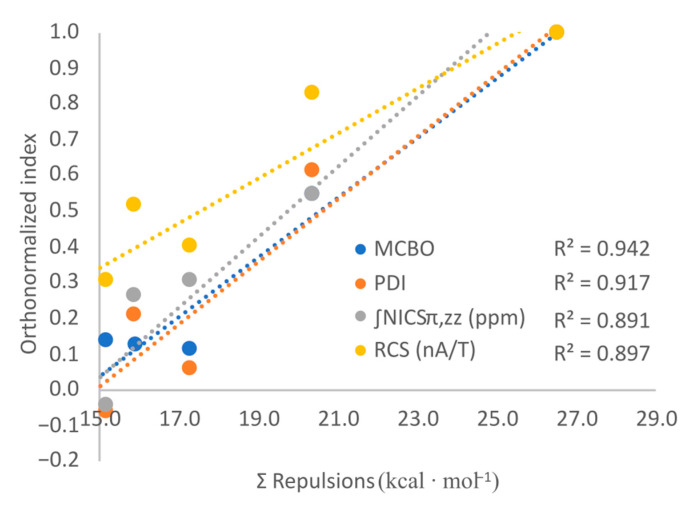
The correlation between the Pauli repulsive interactions and the orthonormalized aromaticity indices for B_3_H_3_N_3_R_3_ (R = F, Cl, Br, OH, NH_2_) models.

By plotting the aromaticity indices as a function of the total repulsion energy (Figure 7), it results in large determination coefficients (e.g., R^2^ values are up to 0.942). Except for the NICS values, the correlation matrices also reveal a strong positive relationship between the aromaticity indices and both conjugative and repulsive effects (see Figures S13 and S14). Yet, by analyzing the corresponding resonance structures resulting from the LP_1_(R)→π*(B=N) donations (Figure 6), it is found that these effects produce electron localization and thus diminish the aromaticity. This suggests that merely the LP_1_(R)↔π(B=N) Pauli effects increase the electron delocalization of such N-substituted borazine systems.

Even though a positive correlation is found between LP_1_(R)→π*(B=N) interactions and the aromaticity indices, this is mainly because conjugations and repulsions increase simultaneously, e.g., the closer two atoms are, the greater the conjugation, but also the steric-exchange interaction. The manner in which such Pauli repulsions increase the electronic delocalization on the ring is achieved by pushing the electron density from the N atoms to the B ones, which leads to more delocalized π(B=N) orbitals than in borazine (i.e., it must be recalled that in B_3_N_3_H_6_, these orbitals are located mostly on the N atoms), and consequently boosts aromaticity. By comparing the computed aromaticity indices (Figure 5) and the total repulsion and attraction energies (Table 2), it is found that the aromatic character increases with the magnitude of both these secondary electronic effects. However, electron conjugations would likely localize the electron density on the ring, which is expected to diminish aromaticity. The same is observed for the field/inductive effects which are also expected to favour the electron localization, through -I attraction. Therefore, it can be concluded for the N-substituted borazines that their aromaticity is mainly impacted by the Pauli repulsions and only to a lesser extent by LP_1_(R)→π*(B=N) donations or by through-space effects.

For the B_3_H_3_N_3_R_3_ systems containing -E substituents, the electron-donor interactions between the π(B=N) bonds on the ring and π* orbitals on the CN or NO_2_ substituents are the most relevant electronic effects. Unlike in the B-substituted analogues, for the N-substituted systems these conjugations are of much higher magnitude, e.g., the computed energy of the π(B=N)→π*(C≡N) interaction is ca. 35.4 kcal mol^−1^/CN group, while for the π(B=N)→π*(N^+^=O) effect, it is 35.8 kcal mol^−1^/NO_2_ group. Differences between the interaction energies of the two model systems can be explained in terms of increased orbital overlaps for the N-substituted borazines, given that π(B=N) orbitals are located mainly on the N atoms and thus the π→π*conjugations with the CN and NO_2_ groups are favoured. These high-energy π→π* donations can explain the decrease in aromaticity of B_3_H_3_N_3_(CN)_3_ and B_3_H_3_N_3_(NO_2_)_3_ compounds compared to borazine since they will favour a partial exocyclic charge localization and thus hamper the electron delocalization on the ring.

The influence of inductive and field effects on the aromaticity of the N-substituted systems is further assessed (Tables S2, S3, S7 and S9 and Figures S2, S7, S9, S15 and S18). As in the case of the B-substituted analogues, this is achieved through the protonation of the OH and NH_2_ groups of B_3_H_3_N_3_(OH)_3_ and B_3_H_3_N_3_(NH_2_)_3_ structures, and the subsequent optimization. As expected, in the equilibrium geometry of the [B_3_(OH_2_)_3_N_3_H_3_]^3+^ system, the s/p LP on the O atom (i.e., LP_2_(R)—the one remaining after protonation) lays in the same plane as the B_3_N_3_ ring, thus leading to an orthogonal arrangement between the LP_2_(R)and the π*(B=N) orbitals on the borazine cycle. Several other σ(O-H)→π*(B=N) (hyper)conjugation interactions occur but are considerably weak. Their calculated energy is ca. 0.8 kcal mol^−1^/OH_2_^+^ group. The OH_2_^+^ groups afford inductive/field effects, withdrawing the electrons from the borazine cycle to compensate for their positive charge. This affects the electron delocalization on the B_3_N_3_ ring, leading to a decrease in aromaticity. The LP_2_(O)→π*(B=N) conjugation can be forced to occur, by optimizing the [B_3_(OH_2_)_3_N_3_H_3_]^3+^ geometry with several constraints (see Figure S7). In this new system, the electron delocalization is slightly higher than in the previous one. Yet, the rather low-energy LP_2_(O)→π*(B=N) interactions (2.9 kcal mol^−1^/OH_2_^+^ group) are motivated in terms of a lower donor ability of the LP_2_(O) orbital, which contains a large percentage of s-orbital in its composition, while π*(B=N) is mostly located on the B atom. Instead, calculations suggest that in this constrained geometry, the LP_2_(O)↔π(B=N) Pauli repulsions are more relevant than the conjugations, with the calculated exchange energy being ca. 18.9 kcal mol^−1^/OH_2_^+^ group. Regarding the [B_3_(NH_3_)_3_N_3_H_3_]^3+^ model system, in the absence of any LP(N) electron-donor orbitals, the NH_3_^3+^ substituents exhibit merely inductive/field effects, withdrawing electrons from the π(B=N) bonds and lowering the aromaticity with respect to borazine.

Regarding the [B_3_H_3_N_3_O_3_]^3−^ and [B_3_H_3_N_3_(NH)_3_]^3−^ model systems (Figure S7), calculations reveal that these structures are considerably more aromatic compared to borazine (Figure 5, Figures S4 and S9; Tables S3 and S5). In these cases, the variation in aromaticity is explained by conjugative and repulsive electronic interactions only to some extent (Table S7 and Figure S13), while field/inductive effects have a major contribution in dictating the aromatic character of the N-substituted anionic borazine derivatives.

## 3. Computational Details

All structures were optimized in the framework of the Density Functional Theory (DFT), employing the PBE0 [33] hybrid functionals and Ahlrichs’s redefined triple-zeta quality basis set, def2-TZVP [34,35]. Long-range dispersion corrections were not considered herein, although previous computational studies [36] assessing the double (i.e., σ and π) aromatic character of several hexa-substituted borazine systems (e.g., B_3_N_3_Br_6_^2+^, B_3_N_3_I_6_^2+^, B_3_N_3_(SeH)_6_^2+^, and B_3_N_3_(TeH)_6_^2+^) included Grimme’s empirical correction [37] in their DFT calculations. Even though in such hexa-substituted model structures through-space secondary interactions (i.e., long-range) between neighbouring substituents could play an important role in the σ-delocalization phenomena (which most likely occurs outside the borazine cycle), for the trisubstituted borazine models investigated in the current work, due to the lack of secondary interactions between grafted substituents, the influence of empirical dispersion on the computed geometries, energies, and wavefunctions is negligible. Hessian matrices were computed for all of the investigated model systems, to characterize the nature of the stationary points on the potential energy surface, i.e., to assess whether optimized geometries possess negative second-order derivatives of energy with respect to their internal coordinates. All calculations were carried out within the Gaussian09 software package [38], implementing an ultrafine integration grid, while the convergence criteria were set to tight. In several cases, molecular structures characterized by higher-order saddle points were needed, with their geometries being optimized via two different approaches: (i) by imposing several geometrical constraints and employing the same optimization algorithm as for the minima, and (ii) by using the Berny algorithm, which searches for (higher-order) saddle points rather than for minima [39].

Aromaticity was investigated by means of four different indices, two of them being related to the electronic criteria, while the other two are part of the the magnetic criteria. A first electronic index, defined by Giambiagi [40], involves the first-order density matrix from which a three-centre bond order (BO) can be derived, thus allowing the characterization of three-centre bonds:

$$I_{ABC}=\sum_{a\in A}\sum_{b\in B}\sum_{c\in C}{\left(PS\right)}_{ab}{\left(PS\right)}_{bc}{\left(PS\right)}_{ca}$$

where P is the density matrix and S the overlap matrix. This equation can be easily adapted to the case of 6-centre bonds, required for the investigated ring systems:

$$I_{ABCDEF}=\sum_{a\in A}\sum_{b\in B}\sum_{c\in C}\sum_{d\in D}\sum_{e\in E}\sum_{f\in F}{\left(PS\right)}_{ab}{\left(PS\right)}_{bc}{\left(PS\right)}_{cd}{\left(PS\right)}_{de}{\left(PS\right)}_{ef}{\left(PS\right)}_{fa}$$

and by one considering all possible permutations between the atoms, the following equation is obtained:

$$MCBO=\frac{1}{2\cdot 6}\sum_{\hat{P}\left(A,B,C,D,E,F\right)}I_{A,B,C,D,E,F}$$

This electronic index (multi-centre bond order, MCBO) was computed with the aid of the Multiwfn program, developed by Tian Lu [41].

The Para-Delocalization Index (PDI) [42,43,44,45,46], developed by Sola et al., is another well-known computational electronic index used to measure the aromaticity of 6-membered rings. PDI is to some extent similar to MCBO, as both rely on the electron density matrix. The major difference between the two indices is that the PDI is based on the Quantum Theory of Atoms in Molecules (QTAIM), defined by Bader [47,48]. For two different atoms of a molecule (e.g., A and B), the delocalization index between them is calculated through double integration of the exchange–correlation density over the atomic basins:

$$\delta^{\alpha }\left(A,B\right)=\delta^{\alpha }\left(A\to B\right)+\delta^{\alpha }\left(B\to A\right)=-2\int_{A}\int_{B}\Gamma_{XC}^{\alpha ,tot}\left(r_{1},r_{2}\right){dr}_{1}{dr}_{2}$$

Thus, one can obtain a quantitative idea of the electron density shared between the basins of the two atoms. Previous studies on benzene revealed that the delocalization index is larger between para-related atoms than meta-related ones, and therefore, the PDI is regarded as a reliable indicator of aromaticity for delocalized 6-centred ring systems [42,43,44,45,46]. All QTAIM calculations performed herein were carried out within the AIMAll software package (Version 19.10.12) [49]. The wavefunctions subjected to partition and further analyses were previously optimized at the PBE0-DFT level of theory in Gaussian09.

Nucleus-Independent Chemical Shift (NICS) analyses are to this day probably the most used methods to assess the aromatic character of cyclic molecules [50,51]. Developed by Schleyer, this methodology has seen many improvements over the years. For instance, given that aromaticity is a multidimensional propriety, Stanger introduced a new approach involving integrals to assess the variation in height of the NICS function [52]. By using Bohman’s decomposition method of the isotropic magnetic moment into canonical orbital contributions and taking into account only the “zz” component of this tensor, the NICS_π,zz_ aromaticity index is obtained [53]. This approach accounts not only for contributions arising from the π orbitals responsible for the aromaticity of a given ring structure but also for the components that are in the direction of the applied magnetic field. If the NICS_π,zz_ parameter is calculated with an increment of 0.1 Å from the ring centre to a height of 4 Å (perpendicular to the centre), the NICS_π,zz_ = f(h) function is obtained. By integrating this function, the most refined NICS indicator is obtained, namely the ∫$NICS_{\pi ,zz}$ parameter. Even though Stanger proposed for benzene and other organic aromatic rings an exponential fitting for this function, in the current study, we found that for borazine and its substituted derivatives, a third-order polynomial is much better fitted (the obtained coefficients of determination, R^2^, were in most of the cases >0.99).

The NMR data were computed at the DFT level of theory (PBE0/def2-TZVP) within Gaussian09, and the GIAO method was employed [54]. Afterwards, the Natural Chemical Shift (NCS) analysis was applied to obtain the decomposed isotropic magnetic moments, which is included in the NBO 7.0 program. For input generation and data extraction, Stanger’s Aroma program was used [55].

Another approach to investigating aromaticity from a magnetic viewpoint is based on the induced ring current, a phenomenon that occurs in aromatic molecules when introduced in a magnetic field. Sundholm developed the Gauge-Including Magnetically Induced Current (GIMIC) methodology [56,57,58] and afterwards the GIMIC program, a tool that can be implemented with Gaussian09. By using the density matrix, the magnetically perturbed matrices (computed herein via the GIAO methodology in Gaussian09, at the PBE0/Def2-TZVP level of theory), and the basis set information, one obtains the induced ring current. By integrating this current through a plane perpendicular to a bond in the ring, the Ring Current Strength (RCS) index is obtained. The ring currents computed for the investigated systems are illustrated in the Supplementary Materials, along with the integration parameters of the RCS.

The computed values of the aromaticity indices were orthonormalized to facilitate the comparison between different types of indices, which in absolute values can range between 0 and 0.5, for MCBO and PDI; between 0 and −27, for NICS; and between 0.5 and 5.5, for RCS. By orthonormalizing the computed indices of the substituted-borazine systems with respect to those obtained for borazine (for which the 0 value was set as the reference), the variation in aromaticity upon substitution can be easily followed. Orthogonalized indices were calculated with the formula:

$$Orthonormalzed\;index=\frac{\left(value\;of\;the\;index\right)-(the\;value\;for\;B_{3}N_{3}H_{6})}{\left(highest\;absolute\;value\;for\;B_{3}R_{3}N_{3}H_{3}\;or\;B_{3}H_{3}N_{3}R_{3}\right)}$$

for every type of aromaticity index and every system type (i.e., B-substituted or N-substituted borazine systems). This leads to values in the range [−1,1] with positive values suggesting an increase in aromaticity compared to borazine (mostly occurs in the case of B_3_H_3_N_3_R_3_ systems), while negative values suggest a lowering of the aromatic character (mostly for the B_3_R_3_N_3_H_3_ models). Given that NICS indices are always negative for aromatic systems, their computed values were multiplied by −1 before orthonormalization, to follow the positive and negative sign rule stated before.

Natural Bond Orbital (NBO) [59,60] analyses were systematically carried out on the equilibrium geometries of substituted borazine model systems, to better understand the connection between the secondary electronic effects generated by R groups and the electron delocalization on the borazine ring. Both attractive (π-conjugations and σ→π hyperconjugations) and repulsive (Pauli-exchange) interactions were evaluated, with their impact on aromaticity being discussed throughout the text. Donor–acceptor attractions were computed with the aid of second-order perturbation theory analyses [61], while steric-exchange effects were determined within the framework of the natural steric analyses [62,63,64]. All NBO calculations were carried out with the NBO 7.0 program [65] implemented in Gaussian09.

Molecular electrostatic potentials (ESPs) were employed to evaluate the magnitude of the through-space electronic effects induced by the investigated substituents on the borazine ring. Wheeler and Houk developed an additive ESP model that facilitates the thorough examination of such effects [66]. The electrostatic potential functions were mapped on the probability density (standard procedure, isovalue = 0.0004 a.u.), while the graphical representations were generated with GaussView program [67]. The calculated ESP maps of investigated borazine systems are illustrated in the Supplementary Materials, along with a short summary of this methodology.

## 4. Conclusions

Even though the aromaticity of borazine has remained a subject of debate to this day, the current theoretical study brings further clarifications to this issue and allows for a more complex picture of the electron delocalization phenomena occurring within its ring structure. Following a systematic analysis of several electronic (MCBO and PDI) and magnetic (∫NICS_π,zz_ and RCS) aromaticity indices, combined with a careful comparison of their computed values (linear regression and correlation matrices), it is shown that both borazine and its B-substituted (B_3_R_3_N_3_H_3_) or N-substituted (B_3_H_3_N_3_R_3_) derivatives (R = Me, SiH_3_, F, Cl, Br, OH, NH_2_, etc.) exhibit a certain degree of aromaticity. It is also highlighted that the aromatic character of borazine can be modulated by playing with the electronic effects of grafted substituents. Thus, substituents known to exhibit -I or +E effects on benzene can significantly increase the aromaticity of B_3_H_3_N_3_R_3_ models, e.g., the aromatic character of B_3_H_3_N_3_O_3_^3−^ and B_3_H_3_N_3_(NH)_3_^3−^ models is up to three times higher than in B_3_N_3_H_6_, according to the computed MCBO indices. The opposite is revealed for the B_3_R_3_N_3_H_3_ systems, given that grafted R groups generally lower the aromatic character of borazine. The influence of different substituents on the electronic ring delocalization and related aromaticity is quantitatively explained in terms of conjugative (attractive) and steric-exchange (Pauli repulsive) electronic interactions. Inductive/field effects also impact the aromaticity of trisubstituted borazine systems, albeit to a lesser extent in most of the studied cases. According to the NBO analyses, the magnitude of the attractive and repulsive interactions can vary considerably with the type of R group, but also with the position of this substituent (i.e., either on the B or the N atom), thus leading to notable variations in the aromatic character. The LP(R)→π*(B=N) conjugations (R = F, Cl, Br, O, or N) are predominant over the LP(R)↔π(B=N) Pauli repulsions in the case of B_3_R_3_N_3_H_3_ systems, while the opposite is highlighted for the B_3_H_3_N_3_R_3_ counterparts. Although in most of the cases (except for R = SiH_3_) the B_3_R_3_N_3_H_3_ isomer is more stable than the B_3_H_3_N_3_R_3_ analogue, the latter possesses an increased aromatic character, which is primarily dictated by the increased steric-exchange interactions. The higher stability of the B-substituted derivatives is motivated based on increased p→π* conjugation effects and weaker Pauli repulsions, which favour electron localization and preclude the occurrence of aromaticity. Therefore, it is shown that even though the aromatic character of borazine can be enhanced by grafting specific electron-donating substituents (F, OH, NH_2_, O^−^, NH^−^) on the N atoms of borazine, the stabilization due to aromaticity has merely a moderate impact on these systems. On the contrary, it is more feasible to disturb borazine’s “quasi-aromaticity” by replacing the H groups on the B atoms with several other substituents that induce stabilization via secondary electronic effects, such as the LP(R)→π*(B=N) donations.

## Figures and Tables

**Table 1:** Computed total energy for the LP_1_(R)→π*(B=N) conjugations and the LP_1_(R)↔π(B=N) Pauli repulsions in the case of B_3_R_3_N_3_H_3_ (R = F, Cl, Br, OH, NH_2_) systems.

Substituent (R)	Σ Donations (kcal/mol/R)	Σ Repulsions (kcal/mol/R)
F	37.1	8.4
Cl	29.1	7.0
Br	24.2	5.7
conj. OH	45.7	11.2
non-conj. OH	20.3	7.8
non-conj. NH_2_	10.9	6.1

**Table 2:** Computed total energy for the LP_1_(R)→π*(B=N) conjugations and the LP_1_(R)↔π(B=N) Pauli repulsions in the case of B_3_H_3_N_3_R_3_ (R = F, Cl, Br, OH, NH_2_) systems.

Substituent (R)	Σ Donations (kcal/mol/R)	Σ Repulsions (kcal/mol/R)
F	4.1	15.9
Cl	2.7	14.5
Br	1.9	11.6
OH conj.	6.4	20.4
OH neconj.	2.9	17.3
NH_2_ conj.	9.6	26.5
NH_2_ neconj.	1.7	15.2

## Data Availability

Data are contained within the article.

## Supplementary Materials

The following supporting information can be downloaded at https://www.mdpi.com/article/10.3390/molecules29204902/s1: Figures S1–S15; Tables S1–S7; additional computational details and information about GIMIC calculations and ESP maps.
