# Supplementary material for: In-Depth Theoretical Investigations of Borazine’s Aromaticity: Tailoring Electron Delocalization through Substituent Effects
Source: Molecules. 2024 Oct 16;29(20):4902. doi: 10.3390/molecules29204902 (PMC11510063; doi:10.3390/molecules29204902)
Supplement: Supplementary file 1 [file molecules-29-04902-s001.zip › molecules-3244296-supplementary.pdf]

Supplementary Materials

In-Depth Theoretical Investigations of Borazine's Aromaticity: Tailoring Electron Delocalization through Substituent Effects

Alex-Cristian Tomut, Ionut-Tudor Moraru * and Gabriela Nemes *

Faculty of Chemistry and Chemical Engineering, Department of Chemistry, Babeș-Bolyai University, 1 M. Kogalniceanu Street, RO-400084 Cluj-Napoca, Romania; alex.tomut@stud.ubbcluj.ro

* Correspondence: ionut.moraru@ubbcluj.ro (I.-T.M.); gabriela.nemes@ubbcluj.ro (G.N.)

1. Computational details and current density maps—GIMIC

In order to investigate the induced ring current of borazine and its trisubstituted derivatives (both $B_3R_3N_3H_3$ and $B_3H_3N_3R_3$ model systems), the Gauge-Including Magnetically Induced Current (GIMIC) method was employed, using the GIMIC program, developed by Sundholm and collaborators [56,57]. The input files necessary for GIMIC calculations were obtained following previous calculations in the *Gaussian 09* software, which enabled the storage of the necessary information into checkpoint files, e.g., the density matrix (*pop=regular*), the magnetically perturbed matrices (*IOp(10/33=2)*), and the basis set information. The transformation generalized contraction basis set was suppressed by employing the *int=nobasistransform* keyword.

The strength of the magnetically induced current passing through a narrow plane placed on a bond from the aromatic cycle was evaluated with a height of 2.5 A above and below the molecule's plane and a length of 4.7 A for all molecules. In all calculations, 200 grid points were included for the integration. Several planes of different lengths and numbers of grid points were considered, resulting in these listed parameters being the most appropriate ones for the current purposes, and also feasible from a computational viewpoint. The Gauss–Lobato integration method was used to integrate the currents passing through the planes. Moreover, the magnetic field was set to be orthogonal on the molecule's plane, within the z-direction.

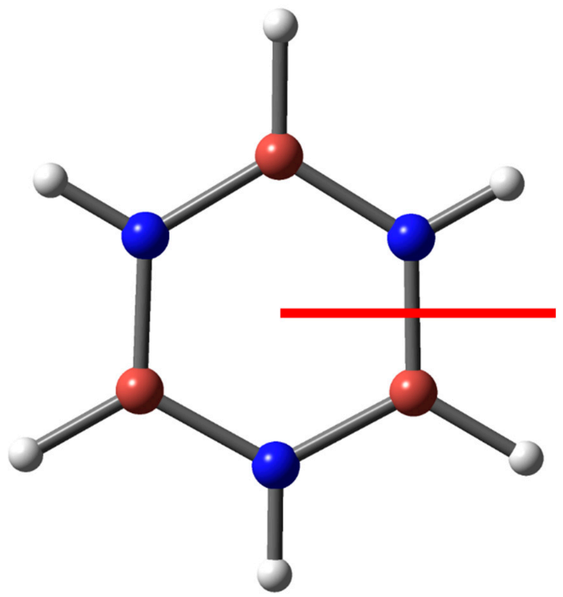

Figure S1. Placement of the integration plane for GIMIC ring current strength calculations (exemplified on borazine).

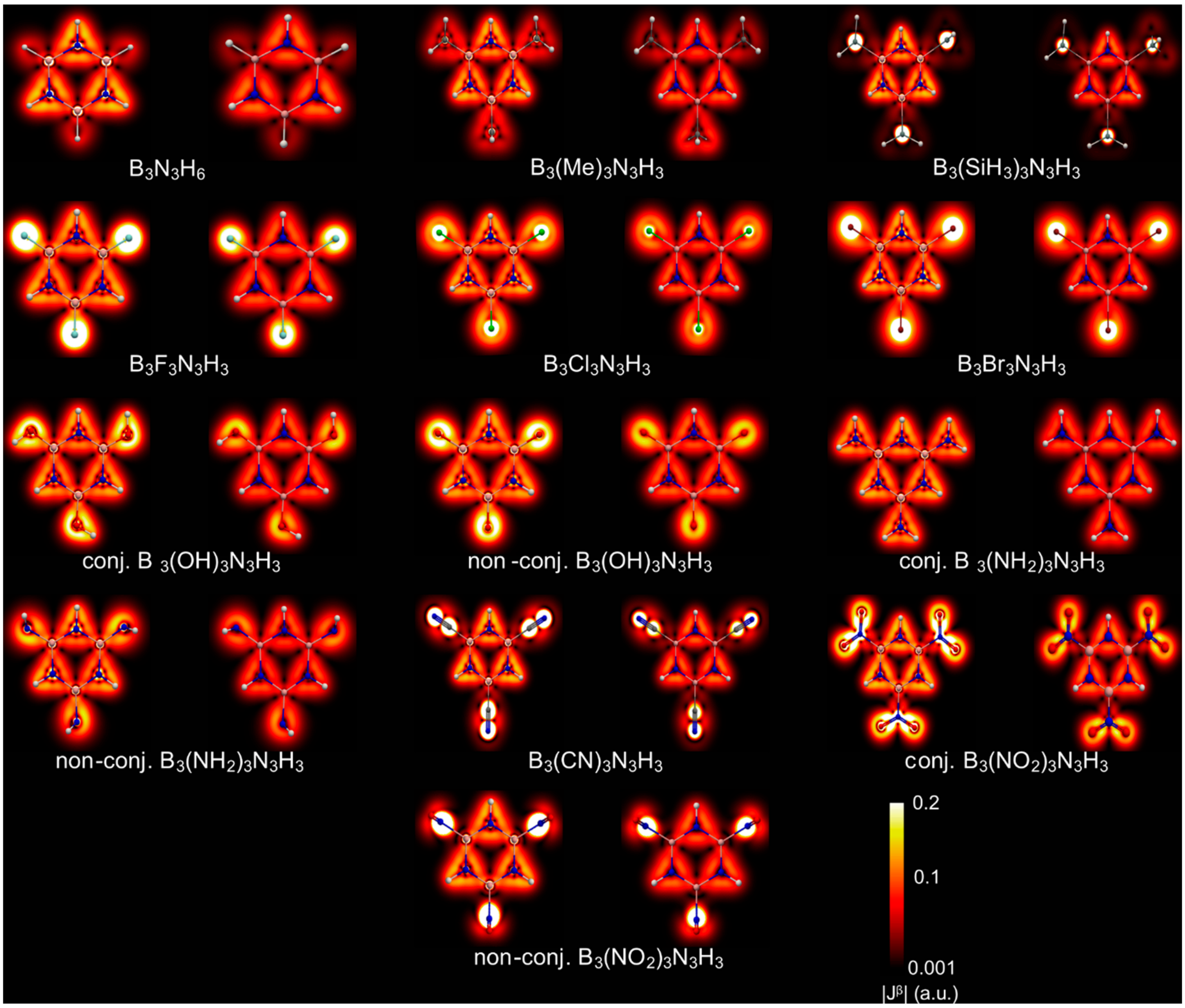

Figure S2. The current density representations at 0 (left) and 0.5 (right) Å above the molecular plane for the substituted $B_3R_3N_3H_3$ systems (1 a.u. = 100.63nA/T/Å^2).

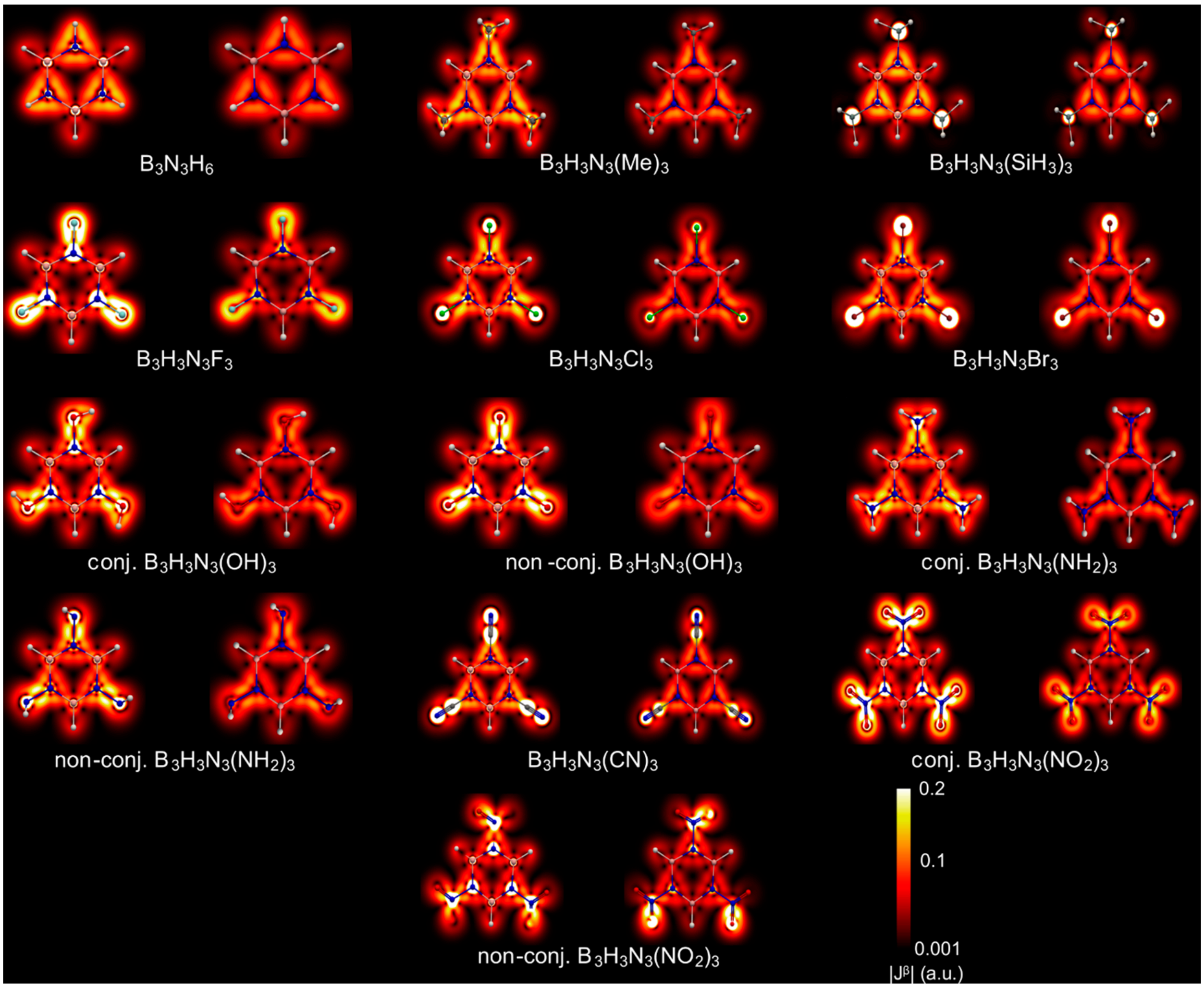

Figure S3. The current density representations at 0 (left) and 0.5 (right) Å above the molecule plane for the substituted $B_3H_3N_3R_3$ systems (1 a.u. = 100.63nA/T/Å^2).

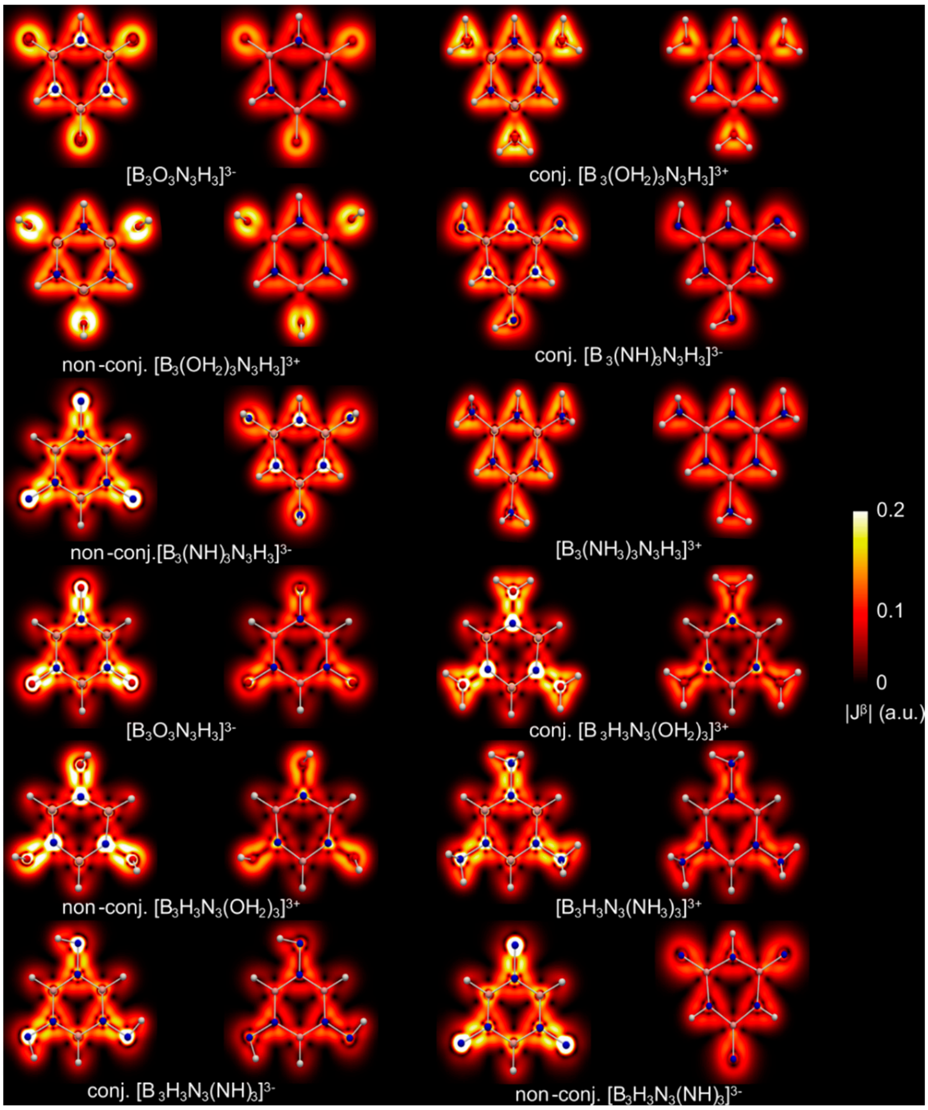

Figure S4. The current density representations at 0 (left) and 0.5 (right) Å above the molecule plane for the substituted charged $B_3R_3N_3H_3$ and $B_3H_3N_3R_3$ systems (1 a. u. = 100.63nA/T/Å^2).

2. Geometrical, energetical, and vibrational parameters

Table S1. The most relevant geometrical and vibrational features obtained for the equilibrium geometries of $B_3R_3N_3H_3$ and $B_3H_3N_3R_3$ trisubstituted borazine systems.

	On B				On N			
**Substituent (R)**	**B-N (Å)**	**B-N-B (°)**	**N-B-N (°)**	**No. of Imag. Freq.**	**B-N (Å)**	**B-N-B (°)**	**N-B-N (°)**	**No. of Imag. Freq.**
H	1.424	122.85	117.14	0	1.424	122.85	117.14	0
Me	1.431	124.10	115.89	0	1.422	120.49	119.51	0
SiH3	1.430	123.76	116.23	0	1.430	119.73	120.27	0
F	1.423	121.24	118.75	0	1.422	127.88	112.11	0
Cl	1.420	121.48	118.51	0	1.429	125.08	114.92	0
Br	1.420	121.29	118.71	0	1.429	124.93	115.08	0
conj. OH	1.429	122.39	117.60	0	1.419	124.33	115.66	3
non-conj. OH	1.429	123.16	116.82	3	1.425	124.90	115.03	0
conj. NH_2	1.435	122.98	117.01	0	1.420	121.79	118.20	6
non-conj. NH_2	1.431	124.21	115.78	3	1.430	122.52	118.21	0
CN	1.421	121.57	118.42	0	1.441	123.53	116.47	0
conj. NO_2	1.408	119.16	120.83	0	1.438	125.16	114.84	3
non-conj. NO_2	1.413	119.48	120.52	3	1.436	125.43	114.58	0

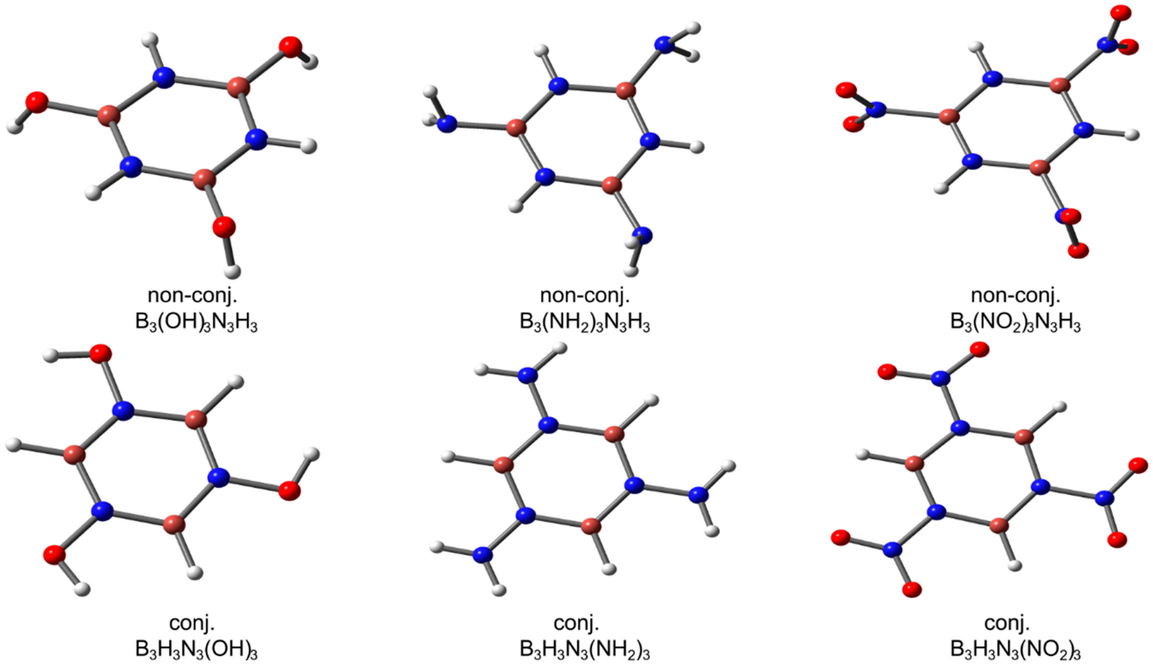

Figure S5. Optimized molecular structures of several higher-order saddle point geometries.

Table S2. The geometrical and vibrational features of optimized substituted charged borazine systems.

Substituent (R)	On B				On N			
	B-N (Å)	B-N-B (°)	N-B-N (°)	No. of Imag. Freq	B-N (Å)	B-N-B (°)	N-B-N (°)	No. of Imag. Freq
H	1.424	122.85	117.14	0	1.424	122.85	117.14	0
O^-	1.484	129.21	110.79	0	1.438	119.17	120.83	0
conj. OH_2^+	1.416	118.42	121.58	0	1.435	127.87	112.13	6
non-conj. OH_2^+	1.413	118.85	121.14	3	1.444	128.63	111.37	0
conj. NH^-	1.474	128.21	111.79	0	1.436	117.73	122.27	3
part.-conj. NH^-		-			1.435	117.96	122.04	0
non-conj. NH^-	1.470	129.23	110.68	3	1.436	118.66	121.34	3
NH_3^+	1.421	120.24	119.76	0	1.437	124.49	115.51	0

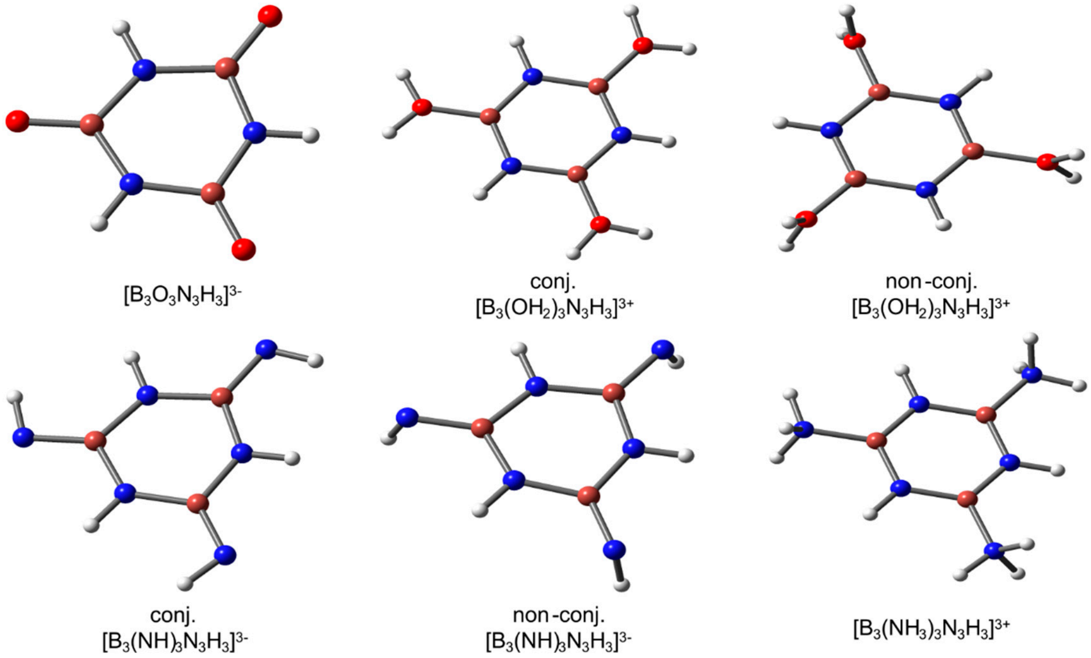

Figure S6. The optimized geometrical structures of investigated cationic and anionic B-substituted borazine models.

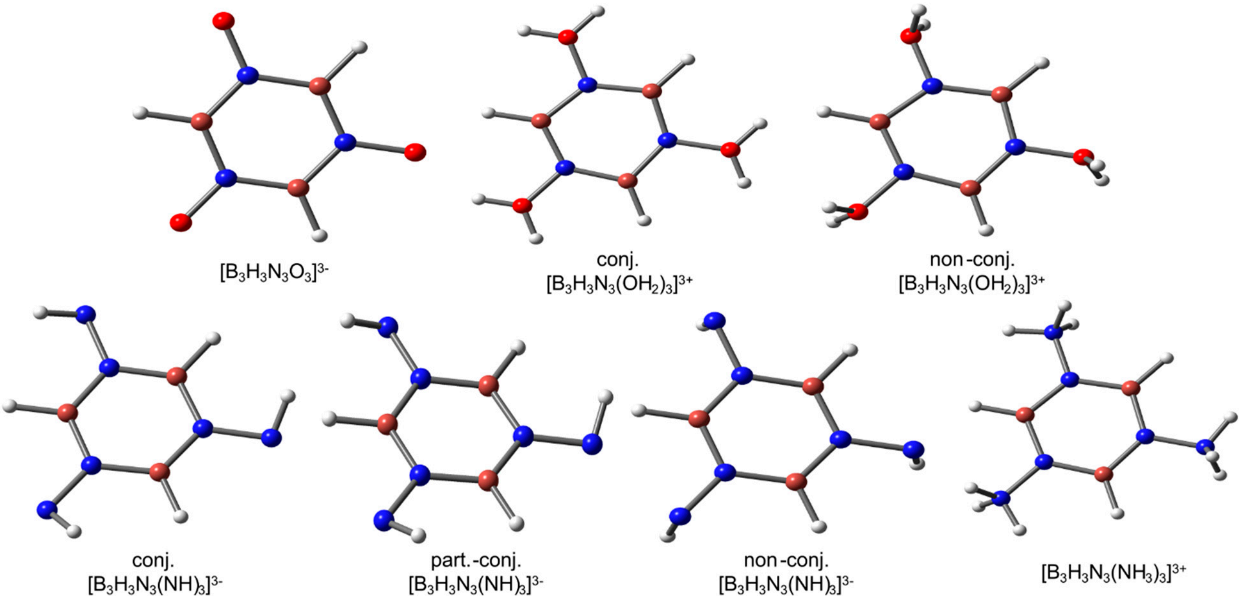

Figure S7. The optimized geometrical structures of investigated cationic and anionic N-substituted borazine models.

Table S3. The relative energies computed for the $B_3R_3N_3H_3$ and $B_3H_3N_3R_3$ model systems. In all cases, the relative energies were reported with respect to the most stable isomer.

Substituent (R)	Energy difference (kcal/mol)	
	on B	on N
Me	0.0	55.9
SiH_3	35.1	0.0
F	0.0	296.9
Cl	0.0	192.7
Br	0.0	169.4
O^-	0.0	233.4
conj. OH	0.0	244.3
non-conj. OH	15.3	244.9
conj. OH_2^+	0.0	291.0

non-conj. OH_2^+	4.8	275.3
conj. NH^-	0.0	174.1
part.-conj. NH^-	-	176.0
non-conj. NH^-	27.8	175.6
conj. NH_2	0.0	196.8
non-conj. NH_2	29.3	177.5
non-conj. NH_3^+	0.0	180.6
CN	0.0	76.4
conj. NO_2	0.0	117.9
non-conj. NO_2	7.1	118.8

3. Aromaticity indices and correlations

Table S4. Computed aromaticity indices for the B-substituted $B_3R_3N_3H_3$ systems.

Substituent (R)	MCBO	PDI	∫NICSπ,zz (ppm)	RCS (nA/T)
H	0.0205	0.0164	-16.96	3.028
Me	0.0160	0.0131	-13.05	2.269
SiH_3	0.0197	0.0238	-15.69	3.023
F	0.0100	0.0093	-8.05	1.648
Cl	0.0130	0.0122	-9.50	1.898
Br	0.0142	0.0135	-9.27	1.890
O^-	0.0056	0.0050	-2.49	0.612
conj. OH	0.0072	0.0078	-6.42	1.142
non-conj. OH	0.0126	0.0102	-	1.927
conj. OH_2^+	0.0102	0.0112	-10.52	2.371
non-conj. OH_2^+	0.0204	0.0164	-17.56	3.332
conj. NH^-	0.0044	0.0049	-1.23	0.380
non-conj. NH^-	0.0119	0.0083	-	1.330
conj. NH_2	0.0056	0.0070	-3.11	0.771
non-conj. NH_2	0.0171	0.0125	-12.96	2.685
non-conj. NH_3^+	0.0174	0.0149	-14.80	2.838
CN	0.0181	0.0149	-15.33	2.909
conj. NO_2	0.0211	0.0163	-17.54	3.232
non-conj. NO_2	0.0147	0.0134	-11.84	2.310

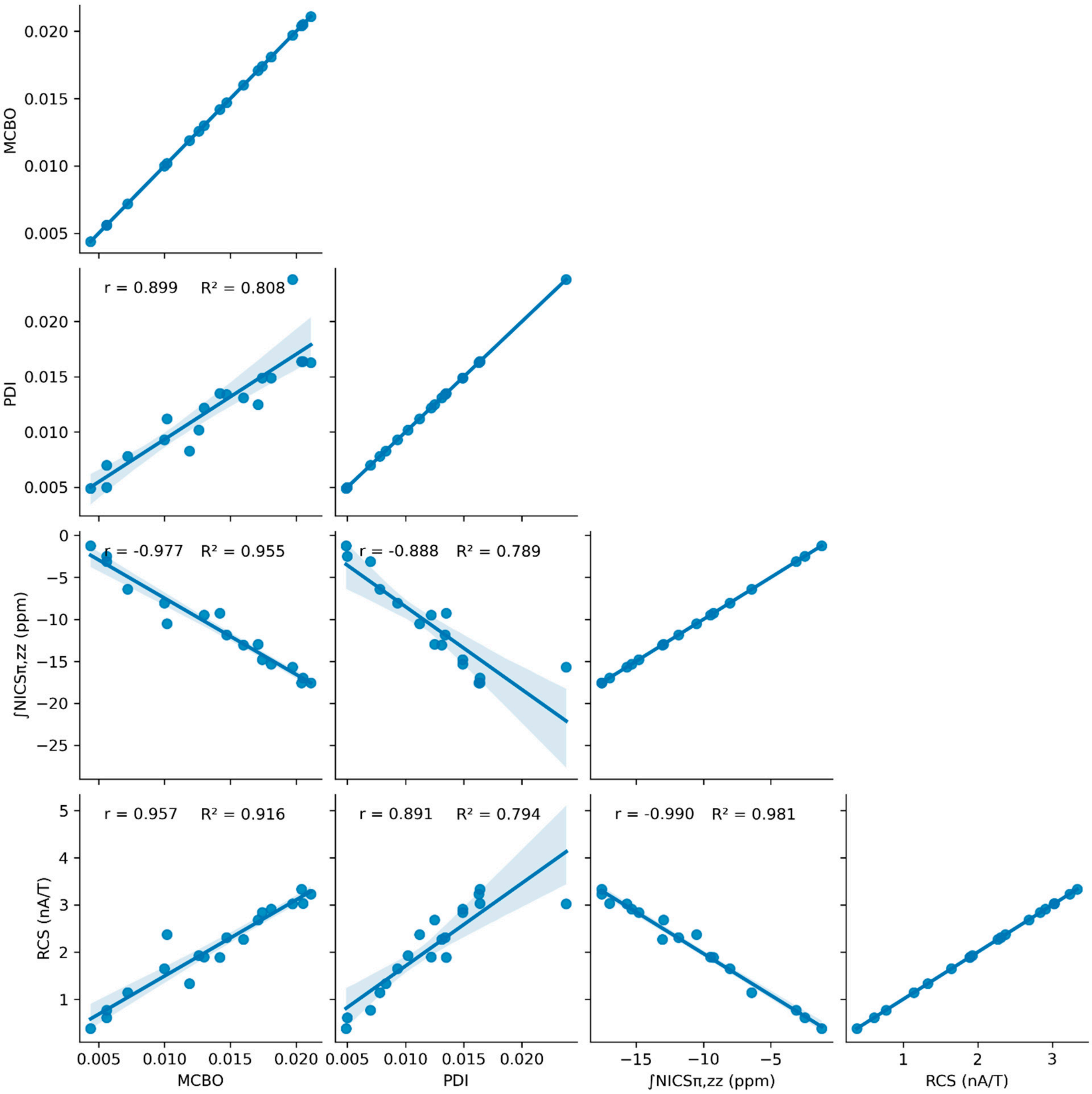

Figure S8. Correlation matrix of the computed aromaticity indices for B-substituted $B_3R_3N_3H_3$ systems.

Table S5. Computed aromaticity indices for the N-substituted $B_3H_3N_3R_3$ systems.

Substituent (R)	MCBO	PDI	∫NICSπ,zz (ppm)	RCS (nA/T)
H	0.0205	0.0164	-16.96	3.028
Me	0.0227	0.0170	-16.71	3.595
SiH_3	0.0151	0.0145	-11.72	3.056
F	0.0223	0.0178	-19.60	3.863
Cl	0.0182	0.0152	-15.53	3.341
Br	0.0184	0.0153	-14.49	3.283
O^-	0.0586	0.0283	-27.44	5.325
conj. OH	0.0278	0.0205	-22.44	4.379
non-conj. OH	0.0220	0.0168	-	3.682
conj. OH_2^+	0.0150	0.0143	-14.47	2.960
non-conj. OH_2^+	0.0102	0.0112	-10.52	2.371
conj. NH^-	0.0581	0.0290	-22.29	4.620
part.-conj. NH^-	0.0564	0.0276	-	4.635
non-conj. NH^-	0.0490	0.0219	-	4.680

conj. NH_2	0.0339	0.0231	-23.64	4.654
non-conj. NH_2	0.0224	0.0160	-16.53	3.528
non-conj. NH_3^+	0.0122	0.0123	-11.76	2.521
CN	0.0104	0.0110	-9.91	2.299
conj. NO_2	0.0095	0.0102	-8.46	1.716
non-conj. NO_2	0.0106	0.0107	-6.90	1.807

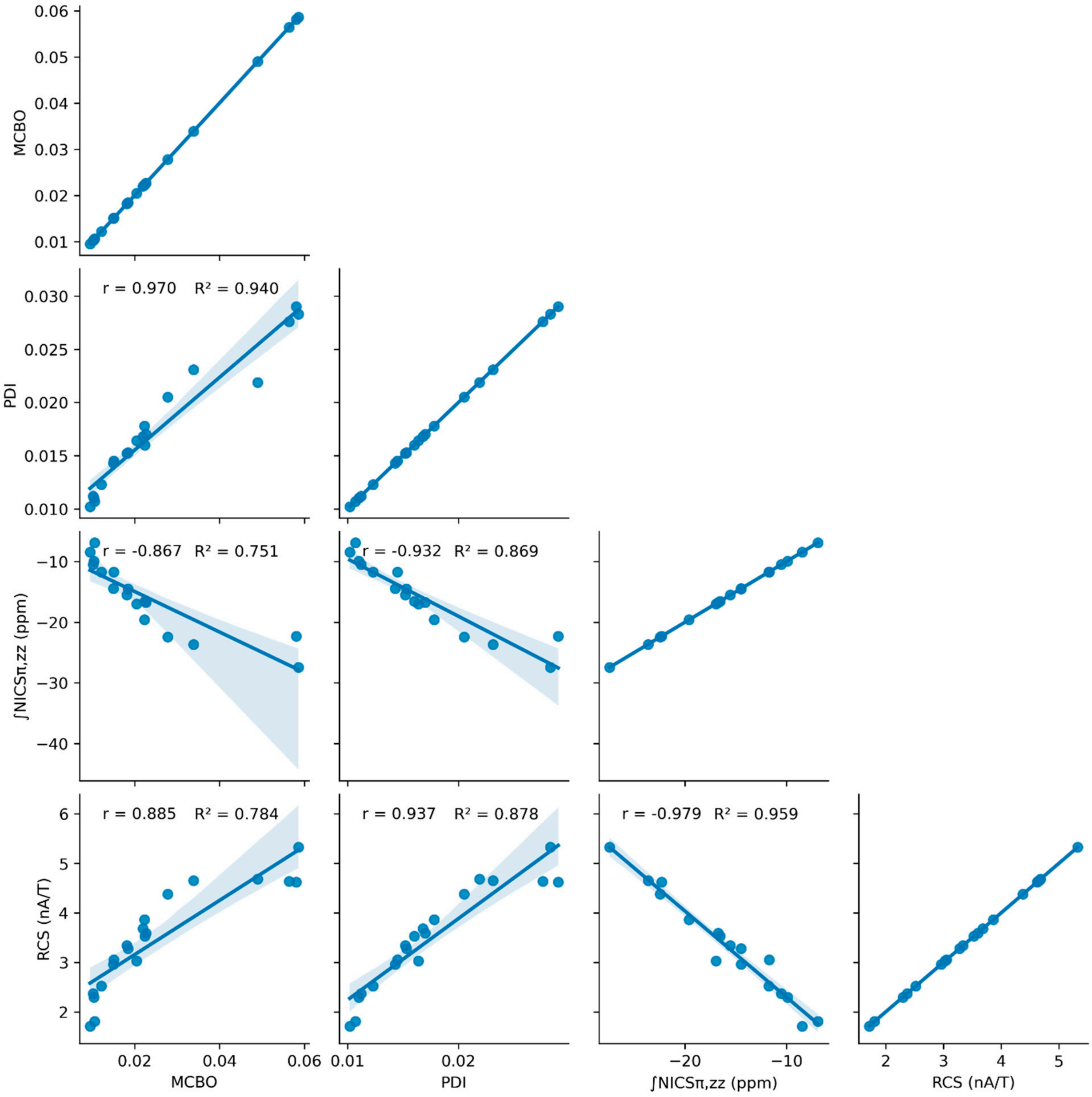

Figure S9. Correlation matrix of the computed aromaticity indices for N-substituted $B_3H_3N_3R_3$ systems.

4. Correlations between the aromaticity indices and electronic effects

Table S6. Computed total energy for the LP1(R)→π*(B=N) conjugations and LP1(R)↔π(B=N) Pauli repulsions in the case of anionic and cationic $B_3H_3N_3R_3$ (R = OH_2^+, NH^-) model systems.

Substituent (R)	Σ Donations (kcal/mol)	Σ Repulsions (kcal/mol)
conj. OH_2^+	23.3	7.37
non-conj. OH_2^+	5.94	4.38
non-conj NH^-	18.2	9.52

Table S7. Computed total energy for the $LP_1(R) \rightarrow \pi^*(B{=}N)$ conjugations and $LP_1(R) \leftrightarrow \pi(B{=}N)$ Pauli repulsions in the case of anionic and cationic $B_3H_3N_3R_3$ (R = O^-, OH_2^+, NH^-) model derivatives.

Substituent (R)	Σ Donations (kcal/mol)	Σ Repulsions (kcal/mol)
O^-	13.15	21.17
conj. OH_2^+	2.97	18.33
non-conj. OH_2^+	0.82	12.28
conj NH^-	14.55	24.68
part.-conj. NH^-	12.43	23.41
fully non-conj NH^-	3.23	18.44

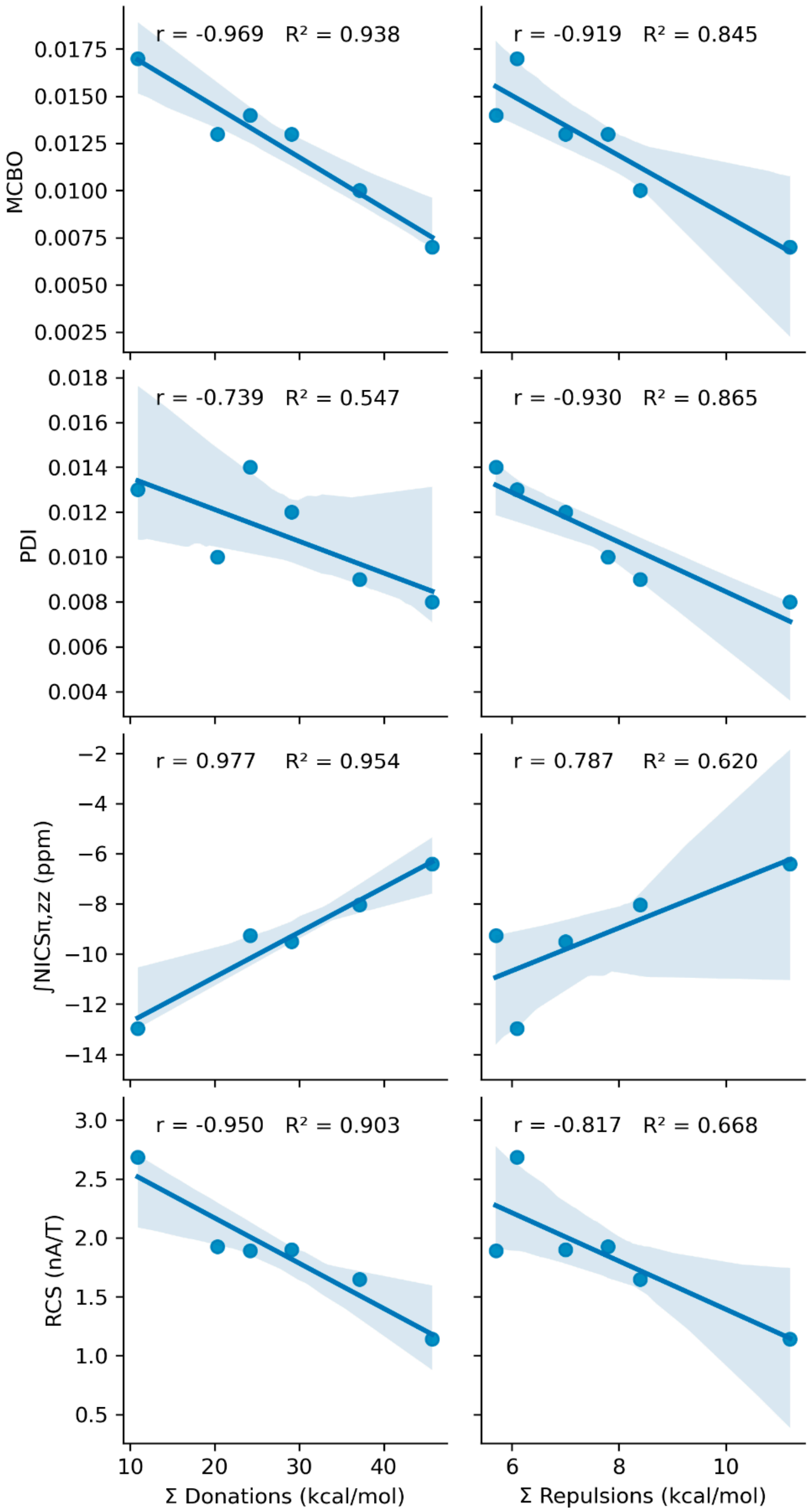

Figure S10. Correlation matrix between the computed aromaticity indices and the calculated energy of donations or repulsions, for the B-substituted $B_3R_3N_3H_3$ systems (R = F, Cl, Br, (non-)conjugated OH, non-conjugated NH_2).

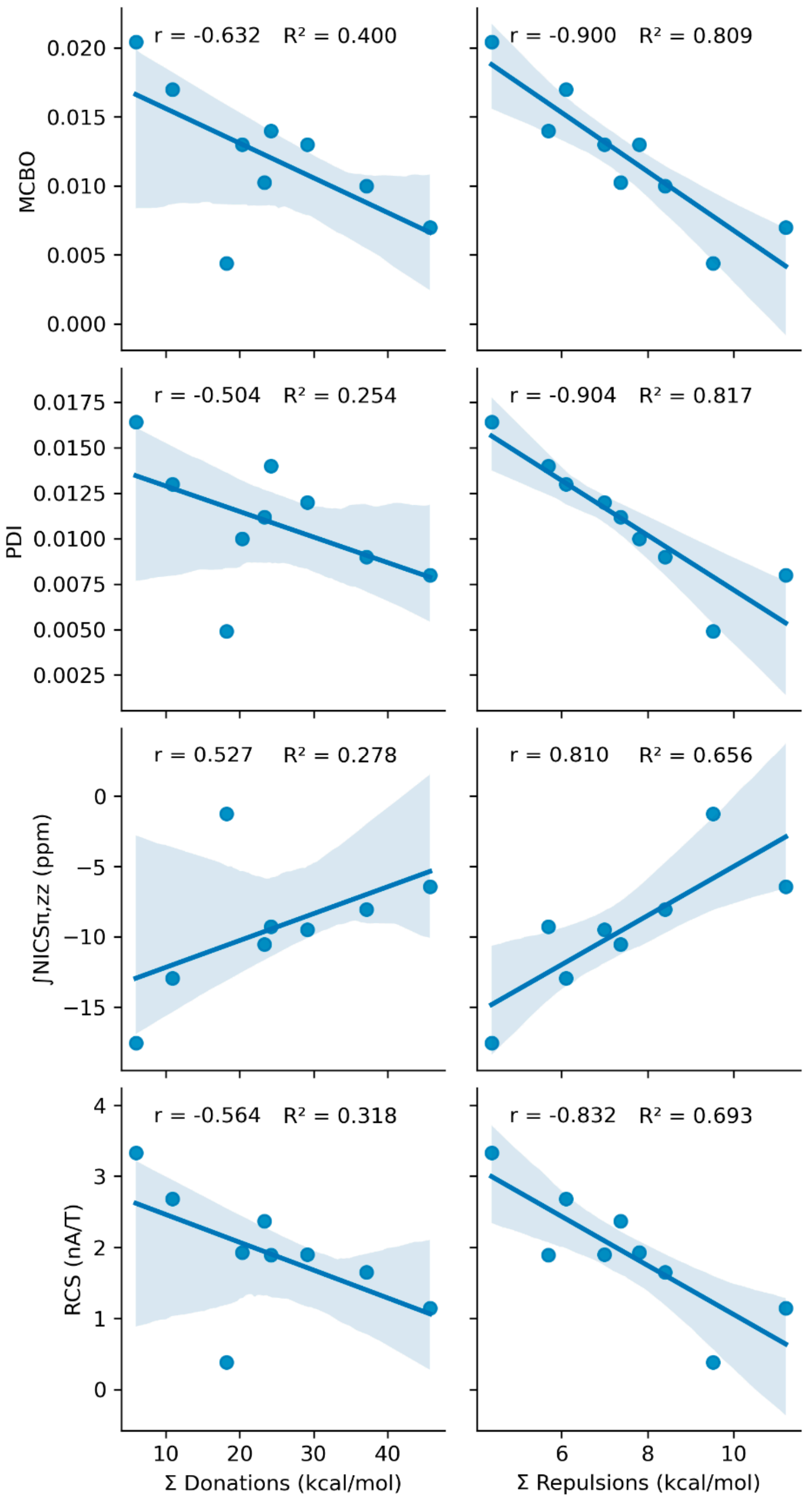

Figure S11. Correlation matrix between the computed aromaticity indices and the calculated energy of donation or repulsion, for the B-substituted $B_3R_3N_3H_3$ systems (R = F, Cl, Br, (non-)conjugated OH, non-conjugated NH_2, (non-)conjugated OH_2^+, non-conjugated NH^-).

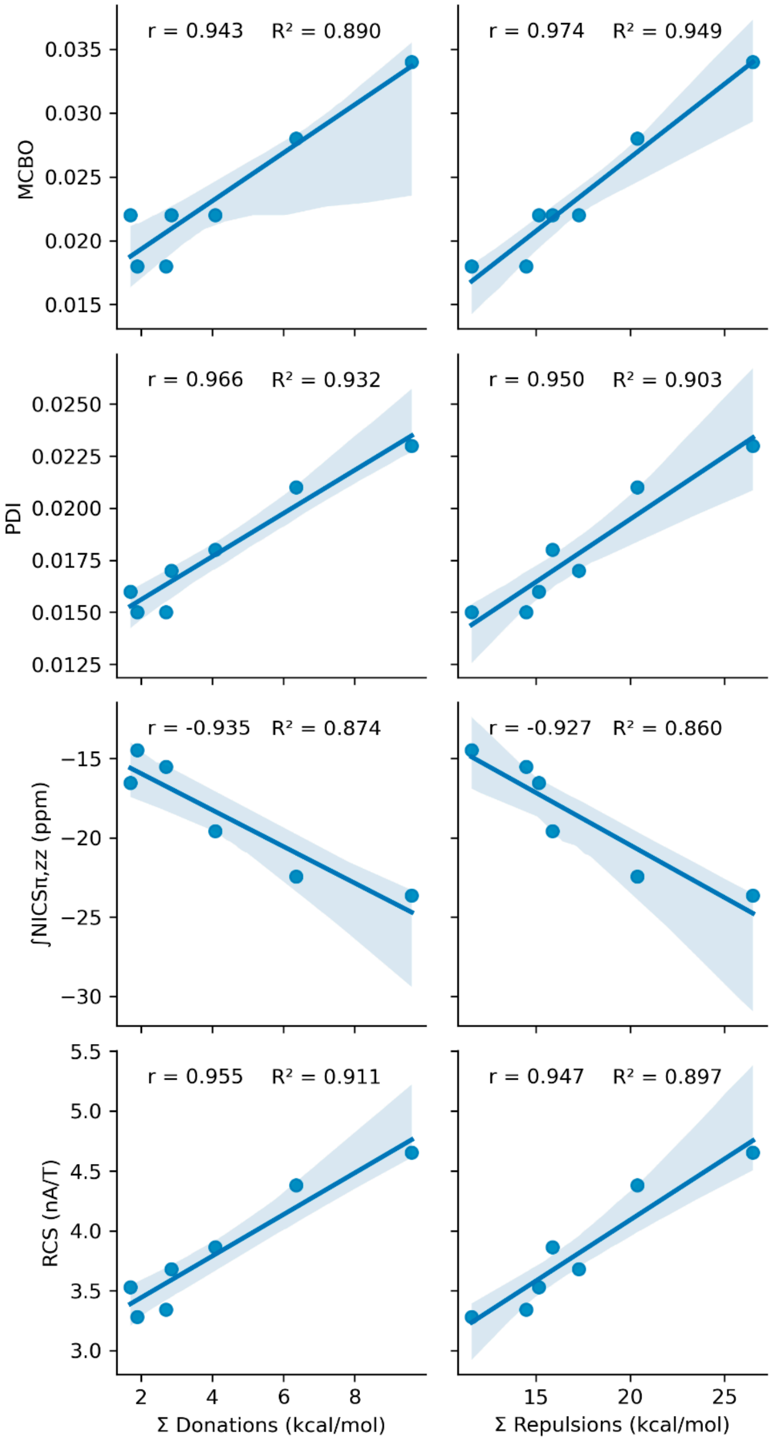

Figure S12. Correlation matrix between the computed aromaticity indices and the calculated energy of donation or repulsion, for the N-substituted $B_3H_3N_3R_3$ systems (R = F, Cl, Br, (non-)conjugated OH, (non-)conjugated NH_2).

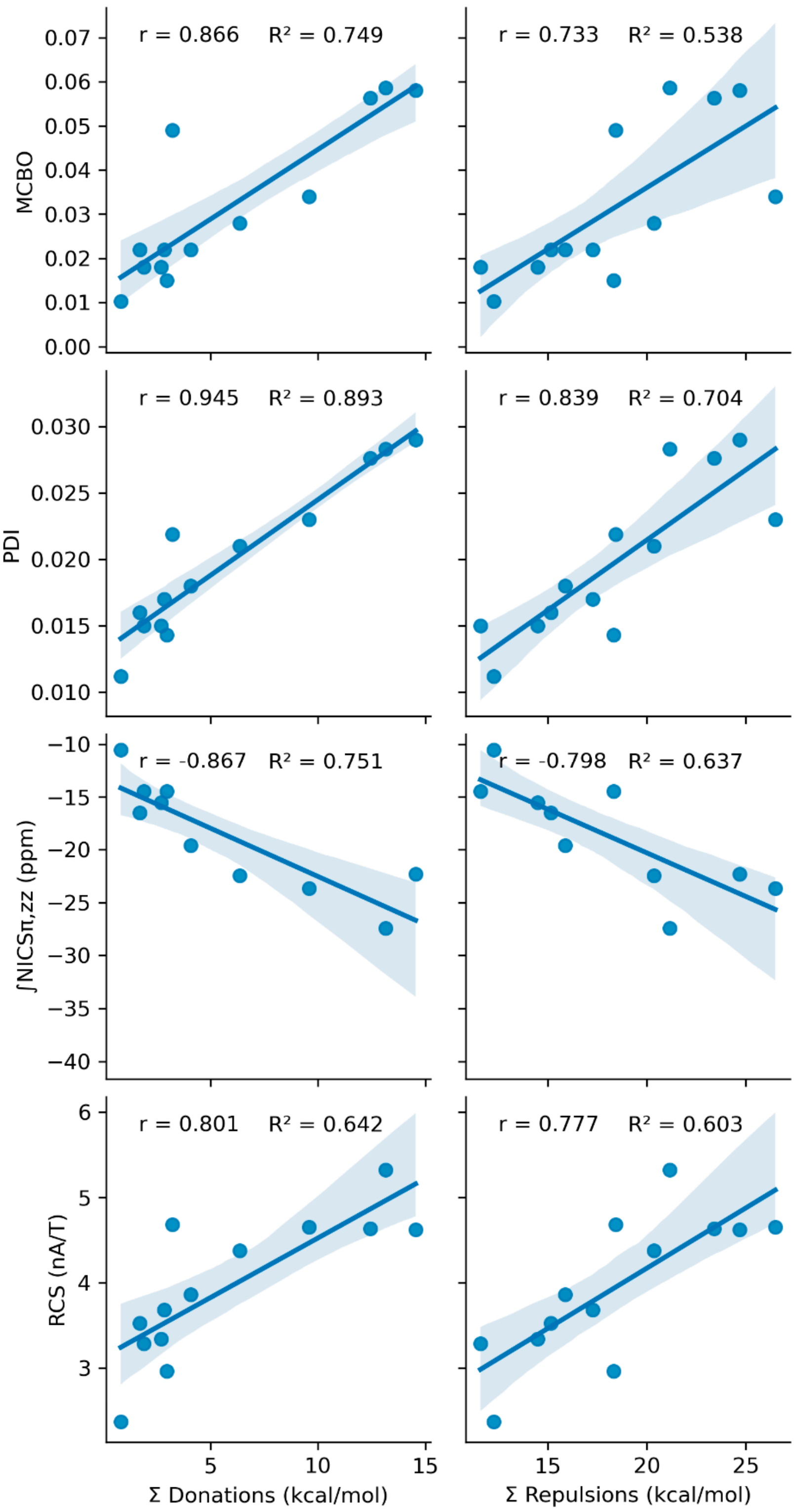

Figure S13. Correlation matrix between the computed aromaticity indices and the calculated energy of donation or repulsion, for the N-substituted $B_3H_3N_3R_3$ systems (R = F, Cl, Br, (non-)conjugated OH, (non-)conjugated NH_2, (non-)conjugated OH_2^+, (part./non-)conjugated NH^-).

5. Electrostatic potential surfaces (ESPs)

The probability density of the studied $B_3R_3N_3H_3$ and $B_3H_3N_3R_3$ model substituted systems was calculated at the DFT level of theory (PBE0 hybrid functionals and Def2-TZVP basis set) within *Gaussian 09* software. The electrostatic potential function was mapped on the probability density (standard procedure, isovalue=0.0004 a.u.), and the graphical representations were generated with *GaussView* tool [67]. For the additive model, the

approach described by Wheeler and Houk was employed. After calculating the density of probability and the ESPs of $B_3N_3H_6$, H-R, and $B_3R_3N_3H_3$, or $B_3H_3N_3R_3$, systems, the ESPs were added to each point with the aid of the *Multiwfn* program and subsequently mapped onto the $B_3R_3N_3H_3$, or $B_3H_3N_3R_3$, probability density [41].

According to the interpretation given by Wheeler and Houk [66], the larger the differences between the standard and additive ESPs, the stronger the conjugative effects (i.e., $p \rightarrow \pi^*$ conjugation; $\sigma \rightarrow \pi^*$ hyperconjugation). According to Figure S14, the strongest interactions are found in systems where R = F, Cl, conj. OH, and non-conj. NH_2, and weak interactions are found in the case of the $B_3(SiH_3)_3N_3H_3$ and non-conj. $B_3(OH)_3N_3H_3$ systems. Interestingly, although the NBO analysis (i.e., E2PERT; see Table 1 in the main text) predicts strong conjugations within the $B_3(NH_2)_3N_3H_3$ model, this is not observed in the additive ESPs. In addition, in line with the paper proposing the model, it is found for the model systems involving CN or NO_2 groups that the field/inductive effects surpass the conjugative interactions. Similar results are found for the N-substituted borazine models, yet the conjugative interactions are considerably weaker, being surpassed by repulsive effects. This behaviour is reflected in the computed ESP maps (Figure S15). The most relevant differences are found for the systems incorporating the F, conj. OH, and non-conj. NH_2 groups, due to the strong donor–acceptor interactions occurring within such structures (Table 2 in the main text). Of course, systems displaying electron-withdrawing groups like CN and NO_2 grafted on N atoms exhibit increased conjugations with the ring, according to the NBO analysis. Yet, as previously shown, these groups afford intense inductive and field effects, while the donor–acceptor phenomena are less meaningful. In the case of the $B_3H_3N_3(SiH_3)_3$ system, it is shown that the ESP map is somewhat different. This is explained in terms of π(B=N)$\rightarrow\sigma^*$(Si-H) hyperconjugations (2 interactions, each of 4.55 kcal mol^{-1}/SiH_3 group) and π(B=N)$\rightarrow$d(Si) back-donations (3.1 kcal mol^{-1}/SiH_3 group), yet the decrease in aromaticity can also be attributed to field effects.

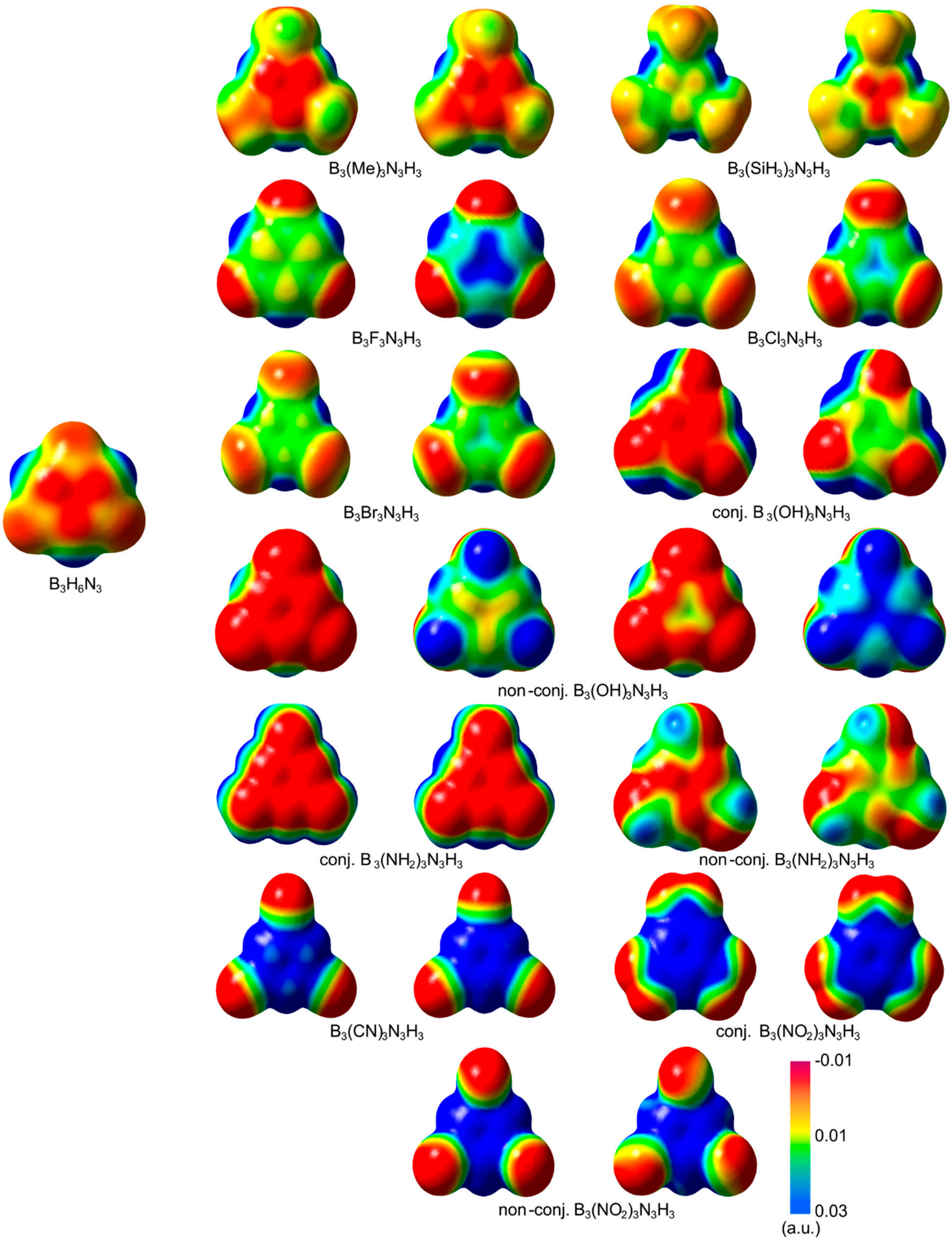

Figure S14. Representation of the electrostatic potential mapped on the probability density using the standard (left) and additive model (right) for substituted $B_3R_3N_3H_3$ systems.

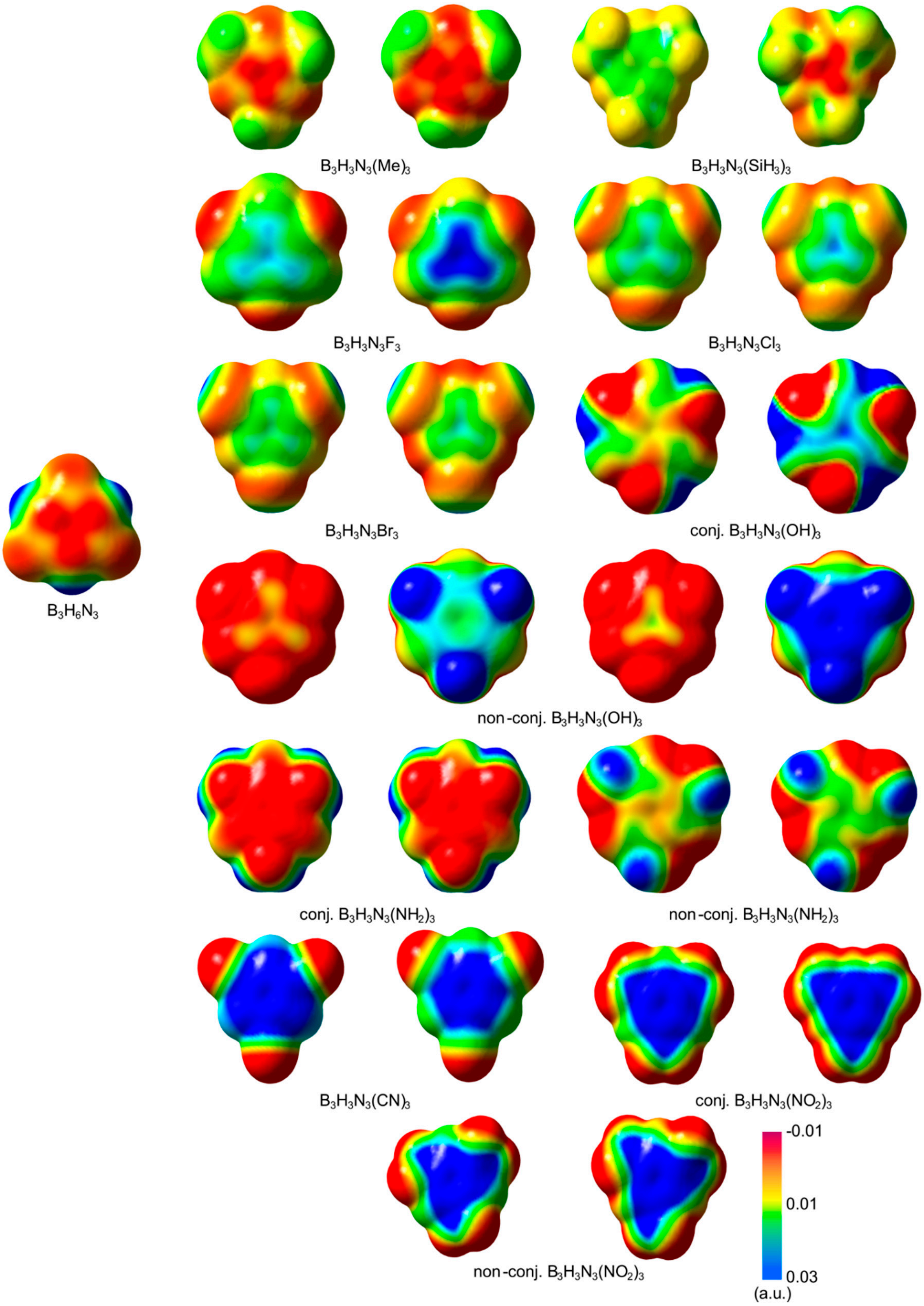

Figure S15. Representation of the electrostatic potential mapped on the probability density using the standard (left) and additive model (right) for substituted $B_3H_3N_3R_3$ systems.
